# "Magic" Ionization Mass Spectrometry

**DOI:** 10.1007/s13361-015-1253-4

**Published:** 2015-10-20

**Authors:** Sarah Trimpin

**Affiliations:** Department of Chemistry, Wayne State University, Detroit, MI 48202 USA; Cardiovascular Research Institute, Wayne State University School of Medicine, Detroit, MI 48201 USA; MSTM, LLC, Newark, DE 19711 USA

**Keywords:** Matrix-assisted ionization, Laserspray ionization, Solvent-assisted ionization, Particles, Clusters, Inlet ionization, Vacuum ionization, Mechanism, Fundamentals, Triboluminescence, Sublimation, Evaporation, Temperature, Pressure, Collisions, Applications, Imaging

## Abstract

The systematic study of the temperature and pressure dependence of matrix-assisted ionization (MAI) led us to the discovery of the seemingly impossible, initially explained by some reviewers as either sleight of hand or the misinterpretation by an overzealous young scientist of results reported many years before and having little utility. The “magic” that we were attempting to report was that with matrix assistance, molecules, at least as large as bovine serum albumin (66 kDa), are lifted into the gas phase as multiply charged ions simply by exposure of the matrix:analyte sample to the vacuum of a mass spectrometer. Applied heat, a laser, or voltages are not necessary to achieve charge states and ion abundances only previously observed with electrospray ionization (ESI). The fundamentals of how solid phase volatile or nonvolatile compounds are converted to gas-phase ions without added energy currently involves speculation providing a great opportunity to rethink mechanistic understanding of ionization processes used in mass spectrometry. Improved understanding of the mechanism(s) of these processes and their connection to ESI and matrix-assisted laser desorption/ionization may provide opportunities to further develop new ionization strategies for traditional and yet unforeseen applications of mass spectrometry. This *Critical Insights* article covers developments leading to the discovery of a seemingly magic ionization process that is simple to use, fast, sensitive, robust, and can be directly applied to surface characterization using portable or high performance mass spectrometers.

**Electronic supplementary material:**

The online version of this article (doi:10.1007/s13361-015-1253-4) contains supplementary material, which is available to authorized users.

**Graphical Abstract:**
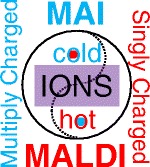
ᅟ

## Introduction

The Taijitu symbol of *yin* and *yang* seems an appropriate representation of electrospray ionization (ESI) and matrix-assisted laser desorption/ionization (MALDI) in mass spectrometry (MS) [1–3]. New evidence lends credence to the idea that these apparently opposing ionization methods, one operating from solution and producing multiply charged ions and the other operating from the solid state and producing primarily singly charged ions, may be complementary methods that, especially for nonvolatile compounds, operate from a common mechanism. Such a possibility was suggested [4], and it now seems possible that ESI and MALDI are special cases of solvent-assisted ionization (SAI) or matrix-assisted ionization (MAI) (Supplementary Table S1), where in SAI the solvent is also a matrix and in MAI the matrix is also a solvent. While the solvation function of a matrix may not require great imagination, the charge separation needed to observe gas-phase ions in the absence of forces such as voltages, heat, or a laser does. This *Critical Insights* is a start in unravelling the magic behind the novel ionization processes for use in MS by showing steps leading to their discoveries and providing suggestions on how they were developed and link with traditional ionization processes.

As was recently pointed out [4], high sensitivity ionization of nonvolatile and high-mass compounds for analysis using MS by any method appears to require a matrix. For the most part, high energy processes such as particle bombardment or laser ablation were used to create the gas-phase ions of large or nonvolatile compounds. However, in 1938, Chapman [5] described charge separation by simply boiling water, and in 1980, Vestal et al. demonstrated that boiling solvents under vacuum produces gas-phase ions from peptides and other nonvolatile compounds for analysis by MS [6]. Ionization of nonvolatile compounds is believed to occur through production of charged solvent droplets [7]. Any method of producing charged gas-phase solvent droplets containing analyte may be used to produce gas-phase analyte ions when the solvent is evaporated from the droplets. Sonic spray ionization (SSI) [8] is one example and SAI [9] is another. The success of ESI is that it is a more efficient means of producing charged solvent droplets, and that of SAI is that a larger fraction of the charged droplets produced within the inlet result in detectable gas-phase analyte ions. Recently, it was demonstrated that combining ESI and SAI, by using voltage to presumably increase the charge on droplets produced within a heated inlet, increased the observed ion abundance relative to either ESI or SAI [10].

Even though charge separation processes are common in nature (e.g., thunderstorms [11]), it was not intuitive that *solid* gas-phase charged particles might also produce gas-phase ions of the analyte with similar charge states to ESI, especially considering the ionization mechanisms proposed for ESI involving, e.g., Taylor cones [12–14]. To my knowledge, no model accounts for the ability of a small molecule matrix compound to produce highly charged gas-phase ions from a solid surface by exposure to vacuum without external energy input (Supplementary Table S1, Supplementary Figure S1, Supplementary Movie Clips) [15, 16]. Under these conditions, the matrix must remain solid so that a mechanism leading to charge concentration, as is proposed for ESI through Taylor cone formation, is unlikely. The method of producing gas-phase ions from a solid, originally termed matrix-assisted ionization *vacuum* (MAIV, Supplementary Table S1), has commonalities with MALDI in that both use small molecule matrices. However, with proper choice of matrix, as discussed below, multiply charged ESI-like ions are produced using an intermediate pressure MALDI source without a laser (MAIV) [15, 16], or with a laser (laserspray ionization *vacuum* or LSIV, Supplementary Table S1) [17, 18]. We hypothesize that the charge separation process produces positively and negatively charged gas-phase particles, or possibly droplets with the use of a laser, which upon loss of neutral matrix molecules by evaporation or sublimation produces the observed analyte ions. A similar process may be applicable in other ionization methods [19, 20] as suggested in the early 80’s [7] for ionization methods available at the time, and later suggested for MALDI [21–28].

## The Need for New Sampling and Ionization Configurations

Beginning in 2004, there was an explosion of novel ionization methods based on known ionization processes of applying high voltages (as in ESI [1] or atmospheric pressure chemical ionization (APCI) [29]) and/or laser desorption (as in MALDI [2, 3] and laser desorption ionization (LDI) [30]). This now decade-old movement to simplify means of ionizing materials for analysis by MS spawned the vigorous field of ambient ionization [31–34]. The success of these developments indicates the need to improve mass spectrometric methodology for increased speed of analysis, ease of use, and higher performance measurements. The first example allowed volatile or nonvolatile compounds with charge states identical to ESI to be analyzed directly from surfaces by desorbing the analytes in an ESI solvent spray, and was termed desorption-ESI (DESI) [35]. ESI was also used as a post-ionization method after, for example, laser ablation of a surface [36–38]. MALDI, LDI, atmospheric pressure photoionization (APPI) [39], APCI [40, 41], and ESI were used in different ways, or hyphenated, in efforts to improve analysis by MS, with some emphasis on surface characterization.

After so many ionization approaches being introduced since 2004, one might think enough is enough; new ionization methods are no longer needed. Such sentiment is not new. As I’ve been told, John Fenn heard similar criticism when he introduced ESI-MS. After all, field desorption (FD) [42], fast atom bombardment (FAB) [43], plasma desorption [44, 45], LDI [46–49], atmospheric pressure ionization (API; now APCI) [29], and electrohydrodynamic ionization [50, 51] were available. Clearly now, as when ESI was introduced, MS is not capable of answering every analytical problem, or in the most straightforward manner, for which it might be capable if there were no restrictions on the ionization processes.

We might also consider if the field is not more likely to be moved forward by new technologies than by continued development (optimization) of mature ionization processes. While there is no assurance that the methods described herein will be the ones that move the field to a new level, I am confident that the insights gained from these processes will play a significant role. In this *Critical Insights*, I attempt to answer questions related to new ionization technology, what these developments may tell us about established ionization technology, and where this might go in the future.

## The Discovery of the Ionization Processes depending on Temperature and Pressure

### Serendipity from a Personal View

It was in 2008, my first year at Wayne State University (WSU), when serendipity played an important role in the unexpected discovery of producing ions with charge states and abundances common to ESI directly from surfaces, by what was anticipated to be an atmospheric pressure MALDI experiment. With startup funds, I wanted to purchase a commercial ion mobility spectrometry (IMS)-MS instrument, but the first generation SYNAPT from Waters would not accomplish the IMS separation my research required. Therefore, I decided to wait for the second generation, but this decision left me without a mass spectrometer in my first year as an assistant professor. Clearly, this was a gamble with my career, but risks are often an important component of success. I subsequently purchased the first SYNAPT G2 to be installed in North America in December 2009. Had I purchased the SYNAPT in my first year, the discoveries outlined below may still be undiscovered. The decision may well have been the best in my career. Fortunately, a number of groups, including Clemmer (IU), Hunt (UVa), McEwen (USciences), and Weidner (BAM), granted us instrument access to bridge this difficult time.

I convinced Chuck McEwen to attempt the transmission geometry experiment described in the Supplementary Information on his newly installed Orbitrap Exactive and newly purchased laser in order to obtain results for my group’s American Society for Mass Spectrometry (ASMS) presentation [52]. Chuck was skeptical of using a laser to ablate a matrix, and potentially tissue, directly into the inlet tube of his brand new mass spectrometer, and argued that transmission geometry atmospheric pressure MALDI had previously been attempted by Galicia, Vertes, and Callahan with ‘disappointing’ results [53]. However, he was kind enough to agree, and had a student, Frank Zydel, set up a makeshift experiment, involving a sturdy table at approximately the height of the mass spectrometer inlet and a stack of papers to accomplish fine laser height adjustments (Figure S1a, Supplemental Movie Clip). This first ‘field free transmission geometry atmospheric pressure MALDI’ experiment produced reasonably abundant analyte ions almost immediately [54]. Galicia and coworkers used a voltage [53], while the experiment that I proposed did not [54]. Therefore, we first assumed that the absence of voltage made the difference. We were able to obtain ions even from mouse brain tissue sections using solvent-based and solvent-free matrix:analyte sample preparation, in which the laser penetrated through the 10 μm thick tissue section from the backside yielding lipid ions [54]. The first ‘transmission geometry mass spectrometry’ images were gratifying [55, 56], however, the big surprise was that ions with charge states and abundances that one associates with ESI were produced from peptides and lipids in a matrix/laser based experiment that, from past training, one would think is MALDI [57]. The term ‘laserspray ionization (LSI)’ was coined to reflect the similarity of the workflow to that of MALDI and the results to that of ESI [58], with potentially a mechanism related to ESI [20].

**Scheme 1:**
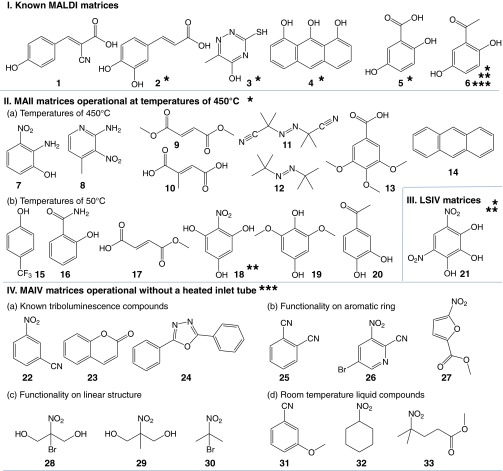
MAI matrix structures indicating general categories for mode of operation: *work with the assistance of higher inlet temperatures without or with a laser, **work with a laser and the assistance of pressure, ***work with the assistance of pressure in the absence of a laser

Serendipity had worked in my favor: I had not purchased a skimmer inlet first generation SYNAPT instrument (Waters Corporation) and had convinced my collaborator with a Thermo Scientific Orbitrap Exactive having a heated inlet tube [59] to help with these initial experiments. Had we used an instrument without a heated inlet tube, the outcome would be different. Experiments on David Clemmer’s IMS-MS instrument without a heated inlet tube failed in the summer of 2009 and when the SYNAPT G2 arrived in December, we were only able to reproduce results obtained on heated inlet tube instruments by using a home-built, add-on inlet tube to the skimmer cone Z-Spray inlet (largely varying in length and diameter, pressures and collisions, Supplementary Figure S1b), but even then only observing ions in relatively low abundance [19, 20, 60–63]. These early difficulties pointed to the need to have a correctly designed inlet and the proper matrix [19, 20, 60–64]. Indeed, the common MALDI matrices α-cyano-4-hydroxycinnamic acid ***1*** (CHCA) (Scheme 1) and sinapinic acid (SA) did not produce multiply charged ions and were later shown to work poorly or not at all, even at 450°C inlet tube temperature on a Thermo mass spectrometer (Figure S1c) [19, 20].

### The Laser is Unnecessary

**Scheme 2:**
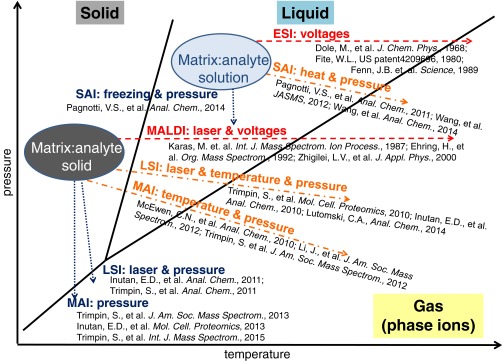
“A phase diagram from a mass spectrometry perspective” illustrating commonalities of the transitions from solid and liquid states to the desired gas-phase ions for use in mass spectrometry by the new ionization process governed by temperature and pressure relative to traditional ionization methods using high voltages and sometimes a laser. A matrix can be a solid or a solvent

The importance of having a healthy dose of skepticism is somewhat obvious, but it is not obvious when to stop forcing results into a familiar box. Chuck McEwen and I fell into this trap and wrote a paper with the catchy title, “An alternative ionization paradigm for atmospheric pressure mass spectrometry: flying elephants from Trojan Horses” [65] in an *International Journal of Mass Spectrometry* issue dedicated to John Fenn. Upon laser ablation, we observed liquefied matrix:analyte droplets using microscopy (Scheme 2; LSI, horizontal transition), and with MS, detected ions from small molecules, peptides, and proteins with charge states very similar to ESI. Therefore, we assumed that charged droplets must be the source of the ions. We also assumed, because we used a laser, that the charges were *initiated* by a photochemical process, similar to what was thought to be the case with MALDI [66]. Thus, based on our trained knowledge (Scheme 2, ESI and MALDI), we had assumed laser ablation leads to charged droplets, which were subsequently desolvated in the hot inlet tube to release the multiply charged ions in a mechanistic process similar to ESI. Although the mechanism still appears to be partially correct, it has now been shown to be incorrect in at least one important aspect: a laser is not necessary and, therefore, photochemical-initiated ionization is not involved in the ionization process [67].

The findings that laser ablation of a matrix:analyte mixture using ultraviolet, visible, and infrared lasers produced nearly identical results [20], and observations of ions after the laser was off were hints that the ions were not produced by a photochemical process. It was only after one of the lengthy discussions between Chuck and me, which can be summarized as ‘*something is not right with our proposed mechanism*’ [65], that matrix:analyte was finally just tapped into the hot inlet of the Orbitrap Exactive (Supplemental Movie Clip). In order to showcase that the laser is not necessary, a BB gun pellet was used to ablate matrix:analyte from a steel plate (Supplementary Figure S1d). Identical mass spectra were produced with and without a laser [67]. Ionization was initiated by charge separation in the inlet using the MALDI matrix, 2,5-dihydroxybenzoic acid (2,5-DHB) ***5*** (Scheme 1) [68]! Of course, past experiments by students that just ‘did not make all that much sense’ kept our imagination active and were important to this discovery.

Interestingly, it was not long after the discovery of “laserless laserspray,” which was termed matrix-assisted ionization *inlet* (MAII) (Supplementary Table S1) [67], and the day the first paper was submitted, we learned that Leonard Nyadong working with Professor Alan Marshall independently discovered the same phenomenon. Ellen Inutan, my first graduate student, and I had previously been invited to the National High Magnetic Field Laboratory to help incorporate LSI onto one of their Fourier Transform (FT) MS instruments. This collaborative work resulted in a publication on the use of laserspray ionization *inlet* (LSII) and MAII for protein analyses from surfaces at the typical ultra-high resolution of their 14.5 Tesla mass spectrometer [69]. This was made possible by implementing a heated inlet tube similar to that previously installed on the SYNAPT G2 and exemplified in Figure S1b [62]. This is the first published example outside of our lab that succeeded in producing highly charged protein ions directly from surfaces using a homebuilt inlet tube for ionization. Importantly, copper cationized analyte ions were observed even without the use of a laser [20, 69], suggesting that the inlet surface is involved in the MAII/LSII process [17, 19, 20, 60–62, 67].

### Functions of the Matrix

Many of our early assumptions, based on past training, did not hold up to experimental verification. For example, we expected that only the DHB isomers that are known to work with MALDI [68, 70, 71] would also work with LSII. Similarly, we initially based matrix selection on those that work using MALDI with the assumption that it would be difficult to discover new matrix compounds. A success story was the finding that the matrix 2,5-dihydroxyacetophenone (2,5-DHAP) ***6*** (Scheme 1) increased ion abundance and required less laser fluence using a commercially available atmospheric pressure MALDI source with the laser alignment in reflection geometry [64]. Later screening efforts of a variety of small molecule compounds at an inlet tube temperature of 450°C demonstrated that many act as LSII/MAII matrices; some are shown in Scheme 1 [18, 19, 64]. These results, as well as others, pointed to the importance of temperature, and as suggested in Scheme 2, also pressure in producing charge separation, which also relates to evaporation or sublimation of the matrix to release gas-phase ions from gas-phase charged particles. The *insight* into the importance of sublimation/evaporation led to a search of more volatile matrix compounds, which would require less heat input to evaporate/sublime from the charged particles/droplets/clusters produced during the charge separation process. It was not clear if charge separation would efficiently occur in the absence of high energy input into the matrix, but because charge separation is common in nature, we had hope.

My group scrounged and purchased a number of small molecule compounds with what we thought were both desirable and undesirable structural features for a matrix. Avoiding carboxylic acid and other functionalities that reduce matrix volatility through salt bridges or formation of salts was one criterion, whereas providing acidic hydrogen atoms through hydroxyl groups was another. We also focused on compounds containing -NO_2_ (as in ‘explosophores’ [72] and plasma desorption matrices [73, 74]) and 1,4-substitution (as in MALDI matrices CHCA ***1*** [75] and SA [76]). Our goal was to understand the structural features of ‘good’ MAII and LSII matrices, and hopefully find some that produced charge separation and were sufficiently volatile for the matrix to evaporate/sublime in vacuum with minimal heat input in order to eliminate the need of a hot inlet tube.

**Figure 1:**
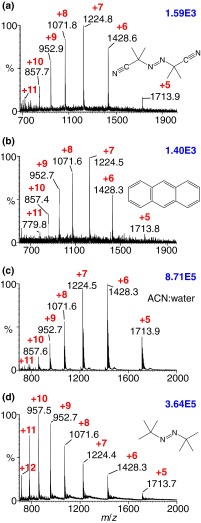
Mass spectra of ubiquitin (MW8560) acquired using (**a**) MAII with 2,2'-azobis(2-methylpropionitrile) ***11*** matrix acidified with acetic acid, (**b**) MAII with anthracene ***14*** matrix in ACN:water acidified with 1% hydrochloric acid, (**c**) SAII (no matrix added to solution), and (**d**) dissolved MAII with 2,2'-azobis(2-methylpropane) ***12*** added into the ubiquitin solution acquired on the LTQ Velos mass spectrometer at 450°C inlet capillary temperature. *Red numbers* indicate the charge states, and *blue numbers* in the upper right corner provide relative ion abundance. Modified from Figures 3 and 6, with permission from Li et al. [19]

Not only did the compounds based on MALDI matrices (e.g., ***2*** to ***6***, Scheme 1) that we expected to act as MAII matrices produce multiply charged analyte ions with a hot inlet tube (450°C), but an equally large fraction of the compounds not expected to be matrix compounds also worked [18–20]. Out of 176 compounds tested at 450°C, 137 provided analyte ions, 56 in high abundance, while 35 gave significant ion abundances at 300°C, 26 at 200°C, and six at 50°C. Importantly, a significant fraction of compounds that did not work or only worked poorly at 450°C or were common MALDI matrices or liquids at room temperature. Examples are CHCA ***1*** and SA MALDI matrices, while caffeic acid ***2*** (Scheme 1), another structurally similar MALDI matrix [76] worked at 450°C. Compounds tested in which carboxylic acid or hydroxyl groups were protected or substituted worked as matrices, especially when the solution was acidified with, e.g., acetic acid (Figure 1a, the 2,2 -azobis(2-methylpropionitrile) ***11***, a matrix that is a known polymer initiator and thermally labile). That acidic hydrogen atoms are not necessary was driven home when it was discovered that anthracene ***14*** (Scheme 1) is a MAII matrix. We found that anthracene (Figure 1b) produces multiply charged protein ions rather well upon addition of HCl, instead of the typical acids used in MS, and produces essentially the same charge states as with solvent-assisted ionization *inlet* (SAII) in which the solvent is the matrix (Figure 1c) [9, 19]. Using the 2,2′-azobis(2-methylpropane) ***12*** matrix from the solution state moves the charge state distribution to lower mass-to-charge (higher charge states) (Figure 1d). Although MAI matrices were found that work well at lower inlet tube temperature, negative mode measurements typically needed higher inlet temperature, resulting in charge states that were lower than those of positive mode measurements [19, 60, 61].

Without the requirement of laser desorption or ablation, no structural features were identified for a compound to be a successful matrix at an inlet temperature of 450°C (Scheme 1). Compounds with acidic hydrogen atoms to protonate the analyte, as suggested to be necessary by at least one model in MALDI [77], are unnecessary, possibly indicating that pre-charged analyte ions reside in the crystallized matrix. Without structural commonalities for the successful matrices, predictions of ‘good’ structural features could not be made. These preliminary results suggest that the most important functions of a matrix are the ability to:

(i) solvate the analyte,
(ii) be involved in charge separation, and
(iii) desolvate the matrix:analyte clusters to obtain the naked analyte ions in the time frame dictated by the mass spectrometer used.

For MAI, the matrix must also participate in the process, whereby the analyte, presumably in a charged matrix particle, is expelled from a surface to the gas phase. A similar process is initiated using an electrostatic potential in ESI, heat and vacuum producing superheating in SAI, and in MALDI ablation caused by conversion of photons to thermal energy by the matrix [19, 20].

### No Need for a Heated Inlet with Laser Ablation

A crucial set of experiments was to test compounds that act as successful matrices at 450°C at a lower inlet temperature in order to allow mass spectrometers without a heated inlet tube (e.g., certain ESI/APCI and vacuum MALDI sources) to be useful with these new ionization processes. At successively lower temperatures, fewer compounds acted as matrices. At the lowest temperature tested, 50°C, most of the compounds failed to produce analyte ions [18–20]. To have a positive spin, we were gratified that now seven matrices (2,5-DHAP ***6***, para-trifluoromethylphenol ***15***, salicylamide ***16***, mono-methylfumarate ***17***, 2-nitrophloroglucinol (2-NPG) ***18***, 1,4-dihydroxy-4,6-dimethoxybenzene ***19***, and 3,4-dihydroxyacetophenone ***20***) (Scheme 1), which produce analyte ions, albeit in relative low abundance, all have in common that they evaporate/sublime under the conditions of the experiment. Again, just as with the 450°C study, a need for certain structural feature(s) is not apparent at 50°C, with the exception that matrices are more volatile. These early experiments lent credence to the proposition that matrix desolvation is a key parameter providing at least a partial explanation of the importance of temperature and pressure in the process of producing gas-phase ions in LSI and MAI (Supplementary Table S1, Scheme 2) [20].

The importance of matrix volatility has been discussed with respect to MALDI [78–80], but not as a means of producing ESI-like charge states. It occurred to us that possibly under just the right conditions, and with one of the matrices that produces ions at low temperature, the energy input from the laser might be sufficient to both produce charge separation and desolvate the charged gas-phase matrix:analyte clusters, thereby producing higher charge state ions than those that are produced by MALDI. Using 2,5-DHAP ***6*** [64], we observed ESI-like ions from vacuum [17]. Here again, we were fortunate that we had an intermediate pressure MALDI ion source (SYNAPT G2) available and observed ‘ESI ions’ from a ‘MALDI’ experiment using a MALDI matrix. Had a MALDI-Time-of-Flight (TOF) been used, ESI-like charge states and abundances would not have been observed with the matrices available at that time (Scheme 1) [54–58]. The observation of highly charged analyte ions from intermediate pressure is believed to be related to the pressure, time, and instrument configuration available for desolvation of charged matrix particles before mass measurement [17–20]. There is no reason to believe that ablation will be any different at intermediate pressure than at the higher vacuum of a MALDI-TOF; however, in MALDI-TOF, the time available for desolvation before ion extraction is in the nanosecond regime [25]. Because no heated inlet is required, as in LSII (Supplementary Table S1), and ionization occurs under vacuum conditions, this method was termed LSIV (Supplementary Table S1), where V stands for *vacuum*.

**Figure 2:**
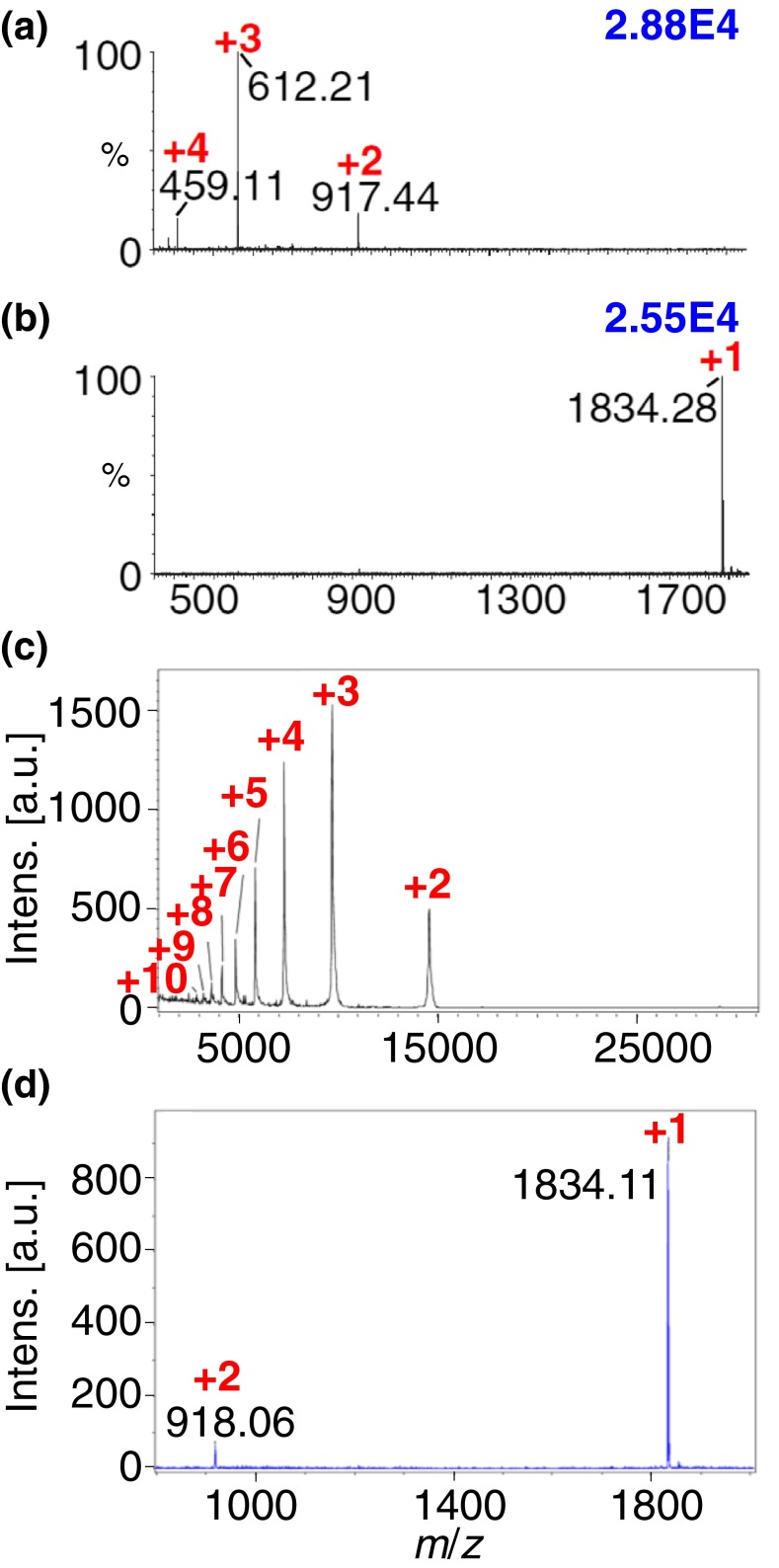
LSIV mass spectra: (top half) *N*-acetylated myelin basic protein fragment (MBP, MW 1833) with 2,5-DHAP ***6*** matrix acquired using the Waters SYNAPT G2 mass spectrometer intermediate pressure MALDI ion source. (**a**) ESI tune, sample plate 0 V, extractor lens 10 V, hexapole bias 10 V, and laser power of 5 J/cm^2^, and (**b**) MALDI tune, sample plate 20 V, hexapole bias 10 V, extractor lens 10 V, and a laser power of 15 J/cm^2^. (Bottom half) (**c**) carbonic anhydrase (MW 29 kDa) and (**d**) MBP peptide prepared using the dried droplet method with 2-NPG ***18*** and acquired in reflectron mode on Bruker high vacuum MALDI-TOF/TOF mass spectrometer. *Red numbers* indicate the charge states, and *blue numbers* in the upper right corner provide relative ion abundance. Modified from Figures 1, 4, and Supplementary S11, with permission from Trimpin et al. [20]

2,5-DHAP is a common MALDI matrix and thus is known to produce predominately singly charged ions [81], but highly charged ions are observed using *low* laser fluence and less energetic conditions (ESI tuning conditions) on an intermediate pressure ion source (Figure 2a) [17, 20]. In contrast, high laser fluence and more energetic conditions (MALDI tuning conditions) exclusively produce singly charged ions from peptides using this matrix (Figure 2b). Thus, 2,5-DHAP is informative of the divide between MALDI and LSIV, one set of conditions producing singly and the other multiply charged ions from the same matrix and analyte. On the other hand, 2,5-DHB ***5***, another MALDI and LSII matrix (Scheme 1) [68], but less volatile than 2,5-DHAP ***6***, does not produce multiply charged ions under LSIV conditions using the same intermediate pressure source. The three matrices that work at intermediate pressure are 2,5-DHAP ***6***, 2-NPG ***18***, and 4,6-dinitropyrogallol ***21*** (Scheme 1). Using these matrices, the formation of ions from small to large nonvolatile compounds [17–20] gives the typical ESI charge states and ion abundance, provided the pressure, voltage, and laser fluence impart low energy relative to typical MALDI conditions. With improved vacuum, the number of operational matrices using this novel ionization process becomes even sparser. The only useful matrix so far found for producing multiple charging with a MALDI-TOF is 2-NPG ***18*** (Scheme 1) [18, 82]. It is noteworthy that binary matrix compositions such as 10:90 2-NPG:SA produce high charge states of ubiquitin from intermediate pressure even though SA alone does not [19, 20]. This is also observed at high vacuum where the binary mixture produced increased charge states of proteins relative to SA alone. Binary matrix mixtures can also be used to lower the inlet temperature requirements on atmospheric pressure mass spectrometers and proved useful for peptide analyses and imaging directly from mouse brain tissue [83, 84].

As noted in the Supplementary Information, experiments demonstrated that upon laser ablation of the matrix, a significant amount of solid matrix is ablated as molten droplets [65, 85]. Ablation of molten matrix upon laser absorption was previously modeled for vacuum MALDI (Scheme 2, horizontal transition) [66]. Therefore, we envisioned that in LSII charged droplets are the precursors of multiply charged ions so that Taylor cone formation could provide the mechanism for production of the small, highly charged droplets envisioned in ESI. The LSIV results, however, posed a challenge because not only did we not have a heated inlet tube, we also needed to use *low* laser fluence (Figure 1a), conditions that seem less likely to form ‘droplets’: higher laser fluence, as noted above, produces lower charge state ions (Figure 1b) [17, 18, 20]. To argue around this apparent glitch, our assumption was that through a thermal process, the laser-generated charged molten matrix:analyte droplets of these volatile matrices are able to desolvate and, upon reaching the Rayleigh limit, generate smaller charged droplets through Taylor cone formation before evaporative cooling stops the process. There were numerous problems with this argument, which were called ‘caveats’ in the initial mechanism paper [20].

The above experiments suggest that the laser ablation process used in MALDI might also produce highly charged particles (clusters or droplets) that, under conditions where they can desolvate, release multiply charged gas-phase ions. Indeed, the 2-NPG ***18*** matrix was discovered to produce multiply charged protein ions on a high vacuum MALDI-TOF mass spectrometer that are stable during flight to the detector [18], contrary to, e.g., CHCA ***1*** (Scheme 1), which also forms multiply charged ions, but these ions tend to decompose during flight [86–90]. If charged clusters are involved, desolvation of the charged matrix clusters, sufficiently large to hold multiple charges, would need to occur in the nanosecond time frame before delayed ion extraction in order to detect ions at the proper mass-to-charge. Desolvation can be enhanced through collisions with objects or gaseous molecules, and possibly by acceleration of clusters through the dense MALDI plume and/or extraction lens of the MALDI-TOF [25]. As noted above, the discovery of LSIV using the intermediate pressure source of a SYNAPT G2 was serendipitous, because the pressure regime and the physical arrangement of this source facilitate desolvation, making it less difficult to produce highly charged ions than is the case for other MALDI sources that operate at lower pressure [18–20, 91–93].

**Figure 3:**
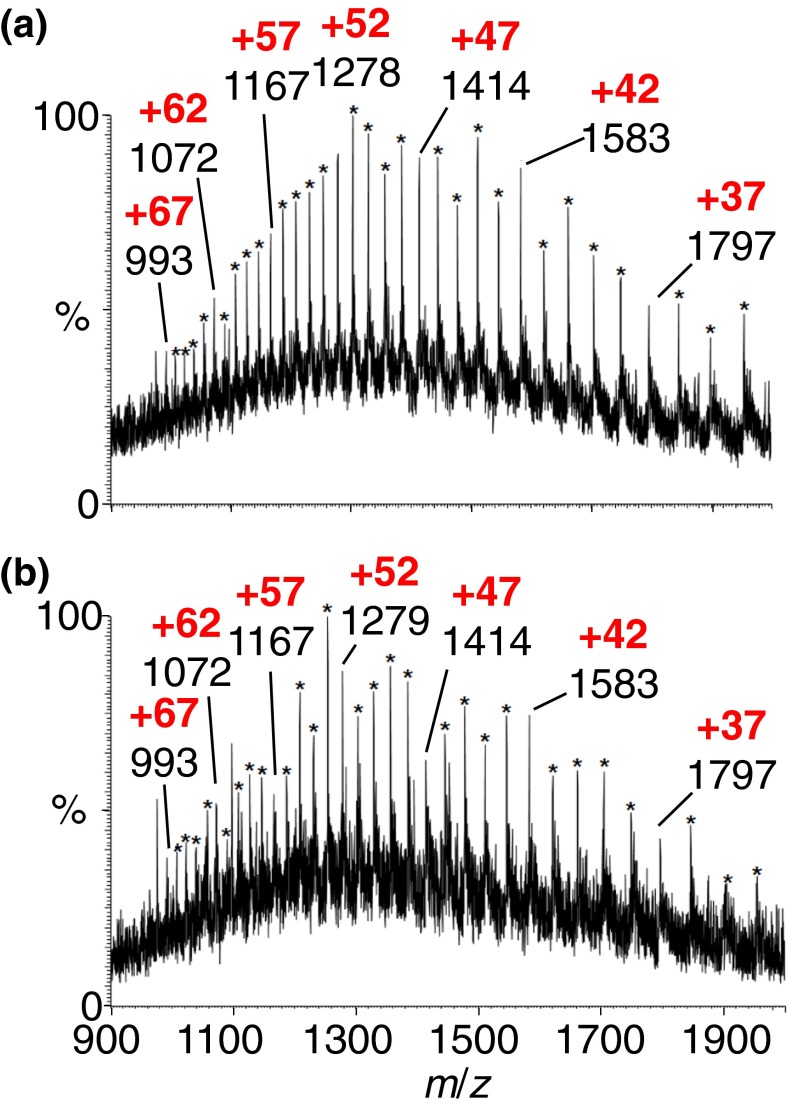
**(a)** LSII-MS and **(b)** MAII-MS spectra of 20 pmol of BSA (~66 kDa) using 2-NPG ***18*** matrix and a 200°C inlet capillary on an LTQ-Velos mass spectrometer. The ion trap was set to 10 microscans and a 100 ms max injection time. The starred and labeled peaks are believed to be the protonated multiply charged protein ions. *Red numbers* and *asterisks* indicate the charge states. Modified from Figure 2, with permission from Lietz et al. [94]

An important point to make is that the higher vacuum (lower pressure) not only makes it more difficult to produce multiply charged ions (Figure 2), but also at the typical high charge states that are observed with LSII and MAII (Figure 3) [94]. Are these clusters simply no longer desolvated in time so that the experiment relies on only the smaller clusters carrying fewer charges and therefore giving fewer analyte charges? Are different sized clusters formed because of the voltages and pressure conditions used, especially in high vacuum TOF mass spectrometers? Murray and Musapelo produced compelling evidence, measuring from atmospheric pressure, that a bimodal distribution of matrix clusters are produced using typical LSI/MAI matrices such as 2-NPG [95–97]. We observed a bimodal charge state distribution in LSIV studies of proteins, especially using the 2-NPG matrix on the intermediate pressure source [20], which might simply be an intriguing coincidence. However, 2-NPG also produces abundant, highly charged ions at atmospheric pressure (e.g., BSA, 66 kDa, +67 charges) with the laser having no identifiable influence on the charge state (Figure 3). Intermediate pressure produces charge states close to those formed from atmospheric pressure but higher protein masses have been difficult even with 2-NPG [18, 20].

The analytical success of LSIV using the matrix 2-NPG is exemplified by imaging of multiply charged ions of a truncated endogenous peptide of myelin basic protein (MBP) ablated from delipified mouse brain tissue as well as other examples [83, 91, 93]. In summary, 2-NPG produces typical ESI-like charge states from atmospheric (Figure 3) and intermediate pressure (Figure 2a); the charge states observed on high vacuum MALDI-TOF mass spectrometers are not observed to the same extent (Figure 2c, d) [18–20, 82, 94]. A typical high vacuum LSIV protein mass spectrum is shown in Figure 2c that has charge states somewhere between those observed with ESI and MALDI; peptides produce few multiply charged ions under these conditions (Figure 2d). Until there are better means for improved desolvation with preferably low energy input (Scheme 2, vertical transition) [82], we may not unravel this problem.

Why peptides have low charge states using 2-NPG matrix from high vacuum conditions is still a mystery (Figure 2d) [20]. Three and four charges are observed on the MBP peptide shown in Figure 2a at intermediate pressure on a SYNAPT G2. Based on the number of charges observed on BSA (Figure 3), it should be readily achievable to desolvate small clusters capable of holding a few charges on a MALDI-TOF mass spectrometer. With atmospheric pressure MAI or LSI, a hotter inlet tube temperature is required for proteins than for peptides [20] but not typically with the matrix 2-NPG (200°C, Figure 3) [60, 94]. Another problem with the desolvation model is the need for lower laser fluence to produce the highest charge states (Figure 2a, c). Thus, a healthy dose of skepticism is warranted, but it may mean that these processes are more complicated than presented above. For example, the high energy and hot matrix droplets resulting from laser ablation may drive loss of ions in the form of protonated matrix or fragment ions, thus reducing the charge on the matrix clusters as previously suggested [98]. Such a process could also explain the observations at intermediate pressure where charge states are reduced at higher laser fluence. Fortunately, the next discovery overcomes some of the unsolved mechanistic arguments, especially the caveat of molten droplets from lower laser fluence conditions (Figure 2a, c, Scheme 2, LSIV vertical transition) [20]. However, the solid state from which ions are formed [15, 16] adds another layer of complexity to understand the mechanistic processes involved as discussed below.

### An Astonishing Discovery: No Need for a Laser or Heated Inlet

**Figure 4:**
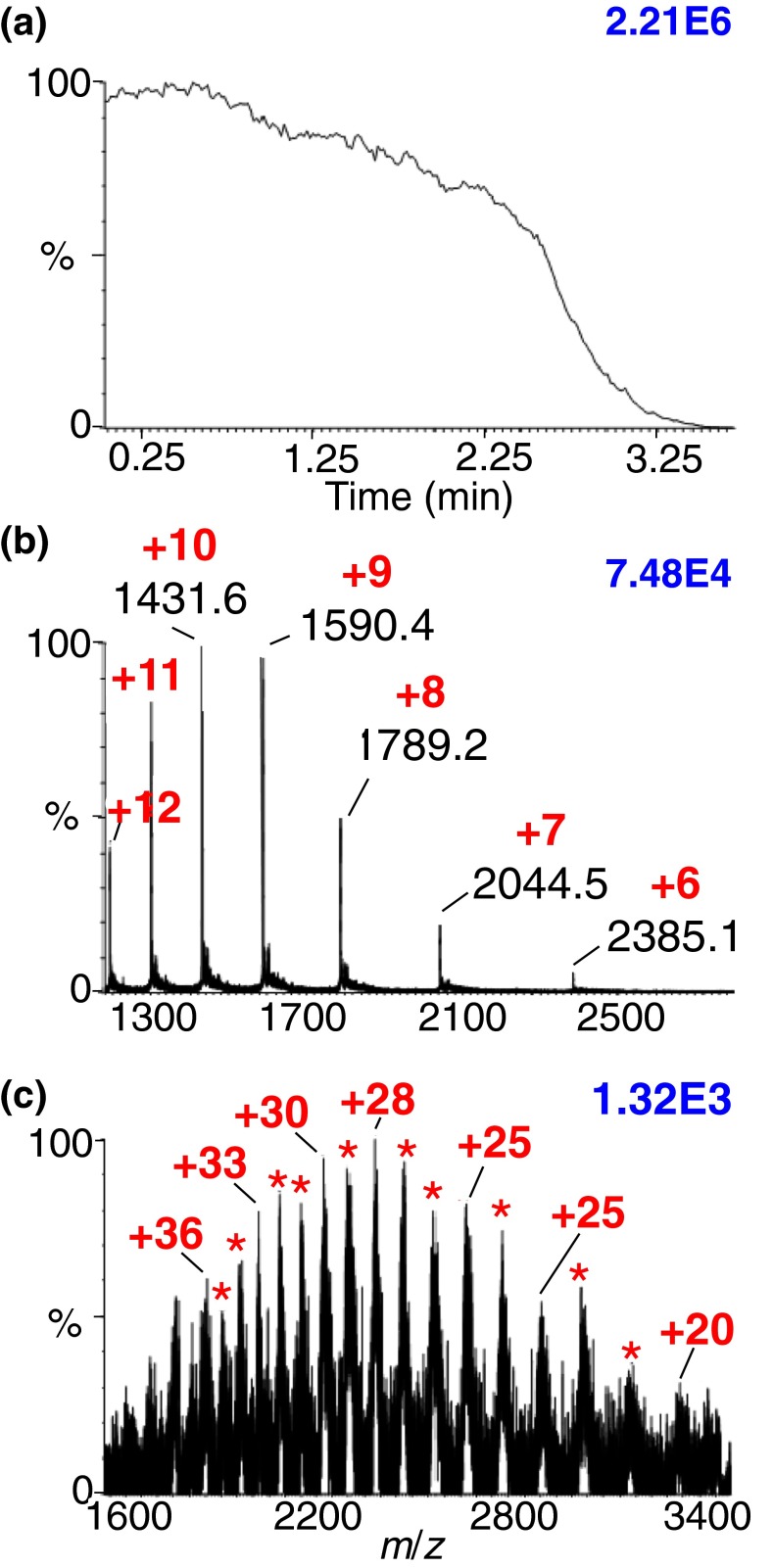
MAIV-MS of lysozyme, 14.3 kDa (**a**) total ion chronogram and (**b**) mass spectrum; (**c**) MAIV mass spectrum of bovine serum albumin, 66 kDa, acquired on an intermediate pressure MALDI source of a SYNAPT G2 *with the laser off* using the matrix 3-NBN ***22***. *Red numbers* and *asterisks* indicate the charge states, and *blue numbers* in the upper right corner of each spectrum provide relative ion abundance. Modified from Figure 1, with permission from Inutan et al. [16]

While it would be nice to claim that the discovery of a matrix that spontaneously produces analyte ions when exposed to vacuum was the outcome of the mechanistic understandings gained in previous studies, it was instead unexpected, even though the matrix had been selected for its volatility. During a lab cleaning day, one of the purchased compounds had not been opened so I asked Ellen Inutan to test this compound using low inlet temperature on the LTQ Velos, as well as LSIV on the SYNAPT G2. Amazingly, this orphan compound not only produced relatively good analyte ion abundance with the LTQ inlet at only 50°C, but Ellen noticed that abundant ions were produced using the SYNAPT G2 intermediate pressure MALDI source even *before* the laser was turned on (Figure 4) [15, 16]! This astonishing discovery is not only analytically useful, but poses a challenge to ionization mechanisms of ESI and MALDI, as well as our own postulates relative to matrix-assisted ionization with and without the use of a laser [20, 65]. We have found no literature precedence that suggests a mechanism whereby gas-phase multiply charged ions are produced without energy input from the solid phase. The process requires a mechanism for charge separation and for producing multiply charged ions of nonvolatile analytes directly from a solid matrix. However, as depicted in Scheme 2 and summarized in Supplementary Table S1, this discovery fills the knowledge gap of the previously described trend in which there is a relationship between the matrix, temperature, and pressure for converting analyte molecules to gas-phase ions.

The discovered matrix, 3-nitrobenzonitrile (3-NBN) ***22*** (Scheme 1) [16], alleviates experimental difficulties described above with the need for heated inlet tubes, as well as those not described, such as instrument contamination with the matrix. This matrix blurs the distinction between MAIV and MAII since it operates in either mode requiring no external force to produce efficient ionization. For simplification, when details are unnecessary, the method is referred to as simply matrix-assisted ionization (MAI) (Supplementary Table S1). Maybe not unexpectedly, the first manuscript on this astonishing spontaneous ionization process was rejected by reviewers of three journals before Michael Gross shepherded it through the *Journal of The American Society for Mass Spectrometry* review process [15]. Although unexpected, this spontaneous ionization does fit with our expectation that matrix evaporation/sublimation (Figure 4a) is an important criterion for observation of analyte ions [20]. 3-NBN readily sublimes when exposed to vacuum (Supplementary Movie Clip), but clearly this is not the only requirement. We found matrices that sublime, similar to 3-NBN [99], but do not assist in forming analyte ions, e.g., 4-NBN [15]. A charge separation process operating from the solid state that also transfers molecules to gas-phase ions is still necessary. During a literature search for properties of 3-NBN, I found that it is known to produce a strong dinitrogen discharge (triboluminescence) when the crystals are fractured [100, 101]. Dinitrogen discharge is believed to occur in the air gap between oppositely charged surfaces of the fractured crystal. Thus, a process that produces 3-NBN crystal fracturing would also produce charge separation. Therefore, a combined hypothesis would require successful MAI matrix compounds to both triboluminesce and sublime when exposed to sub-atmospheric pressure. Because as many as 50% of all organic and inorganic molecules show some degree of triboluminescence [102], it should be possible to find other compounds that work similar to 3-NBN. With these criteria, we quickly found an additional compound, coumarin ***23*** (Scheme 1), which is known to triboluminesce [103], that visually sublimes, and, most importantly, spontaneously produces analyte ions when exposed to vacuum [15].

Based on these findings, a potential mechanism was proposed in which sublimation within defects, or expanding solvent included in the crystals, provide a pressure that produces crystal fracturing, under conditions of surface sublimation, and ejection of both positively and negatively charged particles into the gas phase [15, 16]. Sublimation of these gas-phase charged particles produces the gas-phase analyte ions. The need for triboluminescence and sublimation for MAI is supported by the findings that many matrices that sublime, do not produce analyte ionization, and compounds known to triboluminesce, but do not sublime, also fail to produce analyte ionization [15]. Because MAI is sublimation driven, the process can be sped up using heat [104–106]. However, at temperatures near the melting point of the matrix 3-NBN, the analyte ion abundance greatly diminishes, at least on mass spectrometers without a heated inlet tube [105]; inlet tube sources typically prefer more heat (Scheme 2, horizontal transition) [107].

Obviously, two compounds are not sufficient for any mechanistic argument, but by applying this potential insight and relying on charge separation being common in nature, we have now discovered over 40 of these “magic matrices” for use in MS [106], which should provide a better handle to further elucidate the mechanism of this analytically useful ionization process. Similar to the previous matrix screening results [19], we are unable to identify structurally important motifs [106]. Some of the better and/or structurally different matrices are listed in Scheme 1. Matrix:analyte solutions are typically close to being dry or completely dry but some room temperature solid state compounds work well when introduced to the inlet directly from the solution state, indicating potential applicability with liquid chromatography in the absence of high inlet temperatures. Interestingly, compounds that are liquid at room temperature, although expected to have higher vapor pressure than most solids, failed as MAI matrices despite using high inlet temperatures [19] probably because of the lack of an efficient charge separation mechanism at the temperature employed. However, some of these compounds act as matrices when the temperature is *lowered* sufficiently to convert the liquid into a solid [106]. Similar to the previous matrix study [19], the new low temperature matrices can have linear structures and contain OH groups (Scheme 1). For example, 2-methyl-2-nitropropane-1,3-diol ***29***, carrying exchangeable hydrogen atoms, assists specifically in ionizing compounds that prefer metal cation adduction such as synthetic polymers [108]. Even frozen water (Supplementary Movie Clip), which is known to triboluminesce [109], has been demonstrated to produce multiply charged ions of the analyte [110]. At this time, we attribute the inability of compounds that are liquid at room temperature to perform as MAI matrices, without cooling them to the solid state or heating to reach superheated conditions, to the inaccessibility of a charge separation mechanism under the conditions employed. Little information is available on the compounds discovered so far relative to triboluminescence characteristics, and therefore this is an area that needs further study. Potentially, “cold” spectroscopy and spectrometry approaches may shed light on ionization processes, as is the case with ESI [111, 112]. It is our hope that as we learn more about the charge separation process, this knowledge can be applied to further improve MS ionization methods.

While producing gas-phase analyte ions using a vacuum MALDI source without a laser is mechanistically intriguing, it is not very analytically useful as configured because only one sample can be exposed to vacuum for each analysis [15, 16]. This was an early criticism of some reviewers. Fortunately, the matrix:analyte sample can be introduced to vacuum through the inlet aperture of an API mass spectrometer to efficiently ionize low- and high-mass compounds [104–107]. With low temperature MAI matrices, any type of atmospheric pressure inlet, even without applied heat, provides gas-phase analyte ions for analysis by MS. A variety of different surfaces ranging from glass and metal plates, pipet tips, syringe needles, toothpicks to paper are applicable [15, 16, 104–107]. Movie clips are included in the Supplemental to showcase the simplicity and ease of MAI on different mass spectrometers. Because sample introduction is from atmospheric pressure [104], automation is readily achieved [105]. Rapid analyses are possible because the ionization of a matrix:analyte sample lasts a few seconds at source temperatures of about 50–150°C depending on the source geometry [19, 20, 82, 105–107]. It was also shown that a flow of warm nitrogen gas (~60°C) over the matrix:analyte sample placed on a melting point tube in front of the inlet of a mass spectrometer is sufficient to produce ions from small proteins using 3-NBN and, with additional heat (~100°C), 2,5-DHAP ***6*** as matrix [16, 113].

There seems to be a distinct difference for small and large compounds being ionized by MAI: some small molecules seem to have a rather ‘harsh’ experience even with a near room temperature inlet [114], whereas higher molecular weight compounds seem to enjoy a much ‘softer’ ionization process (Figure 4b, c) [16]. That is, under the same experimental conditions, specific product ions of small molecules are generated to some degree, but no fragmentation is observed for peptides (or proteins) [105]. In a systematic study by Fenner and McEwen of ESI, SAI, and MAI using small compound ‘thermometer molecules’ SAI provided the softest conditions whereas MAI showed the most fragmentation [114]. One possible explanation may be that high-energy photons are produced in triboluminescence, even in the regime of X-rays [115]. When air is present, 3-NBN provides radiation similar to a nitrogen laser because of the dinitrogen discharge [100, 101]. It is conceivable that this ‘built in’ energy source might increase the internal energy of compounds that absorb photons at the emission wavelength. This radiation is unlikely to be involved in the matrix-assisted process that produces gas-phase analyte ions, because the 3-NBN matrix does not absorb at this wavelength [101] nor do a number of the over 40 compounds discovered to act as MAI matrices near room temperature (Scheme 1) [106].

The continuous sublimation of 3-NBN:analyte crystals under intermediate pressure (Figure 4) [16] makes it difficult to interrogate the effect of laser ablation on ionization with this matrix using the SYNAPT G2. In a collaborative effort with Charlie Wilkins’ group, using a high vacuum MALDI source on an FTMS instrument, where sublimation of 3-NBN takes about 30 min relative to about 3 min on the higher pressure SYNAPT G2 MALDI source (Figure 4a), it was shown that identical charge states and nearly the same ion abundances are obtained with and without use of a laser [92]; the typical ultra-high resolution was achieved. This suggests that the laser serves to more rapidly cause the consumption of the 3-NBN:analyte sample but does not have an influence on the ionization event. Interestingly, using the same mass spectrometer and low pressure source but instead the 2-NPG ***18*** matrix, significantly lower charge states were obtained (e.g., for ubiquitin a maximum charge state +4 with the most abundant charge state +2). From past experience, the 2-NPG matrix requires a laser and, as discussed above, ‘only’ extends the formation of higher protein charge states (Figure 2c) on high vacuum MALDI-TOF mass spectrometers [18]. Using the same FTMS instrument, but from atmospheric pressure and using 3-NBN as matrix, the typical high MAI charge states are detected [92]. Future ion source in MS will benefit from improvements in charge separation and desolvation conditions, especially at vacuum [17–20, 60, 61, 82].

### Mechanistic Insights

Our current hypothesis is that when exposed to vacuum, or heat alone, MAIV [15, 16, 106, 113] matrices spontaneously fracture, either from sublimation pressure or pressure from expansion of included solvent, thus expelling charged matrix:analyte particles and clusters into the gas phase, possibly through microexplosions. This process, whereby only a small fraction of the total molecular composition in a particle is charged, is far more energetically favorable for charge separation than direct expulsion of a bare ion. The power of expansion driven processes is seen when, for example, included water is sufficient to cause crumbling of granitic rock during cycles of heating and cooling [116]. Temperature and pressure have roles in both charge separation producing gas-phase charged particles and desolvation of these particles. Once charged particles enter the gas phase, sublimation of the matrix releases analyte ions for analysis by MS*.* If this hypothesis is correct, it means that highly charged gas-phase ions are produced from solid particles, a process that demonstrates a gap in our knowledge, as discussed below.

**Scheme 3:**
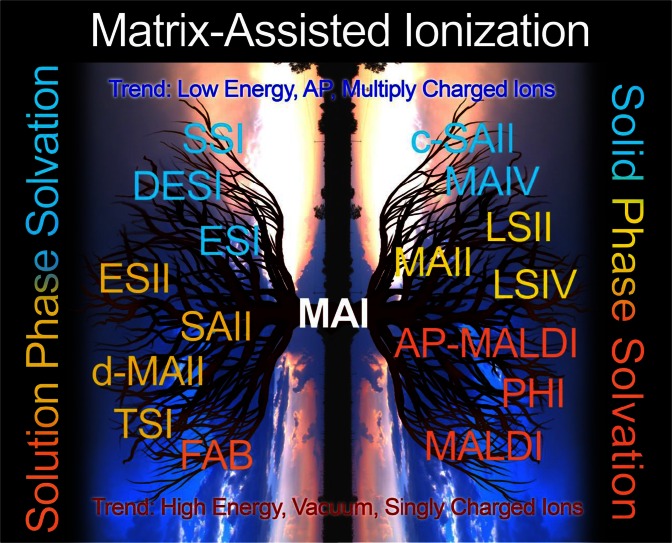
Acronyms of methods used in MS for analysis of nonvolatile compounds shown on the branches of a tree and its reflection. Matrix-assisted ionization (MAI) is shown as the base of the tree. Methods that operate from solution (left) and solid states (right) are organized from bottom to top into hot (light red) to cold (blue) energetic conditions with ionization methods that produce multiple (top) or primarily singly (bottom) charged ions. *Left top to bottom*: sonic spray ionization (SSI), desorption electrospray ionization (DESI), electrospray ionization (ESI), electrospray ionization *inlet* (ESII), solvent-assisted ionization *inlet* (SAII), dissolved matrix-assisted ionization inlet (d-MAII), thermospray ionization (TSI), fast atom bombardment (FAB); *right top to bottom*: cold SAII (c-SAII), matrix-assisted ionization *vacuum* (MAIV), matrix-assisted ionization *inlet* (MAII), laserspray ionization inlet (LSII), laserspray ionization *vacuum* (LSIV), atmospheric pressure (AP) matrix-assisted laser desorption/ionization (MALDI), pulse-heating ionization (PHI), and MALDI

In ESI, Taylor cone formation leads to droplets in which the field strength is sufficient to expel solvated ions directly from the droplet (ion evaporation model, [117]) or to charged droplets that are expelled from Taylor cones in which only a single analyte molecule is present and released as a gas-phase ion upon complete solvent evaporation (charge residue model) [118]. In order to fit the ESI mechanism to a solid charged particle, there needs to be a process whereby charged particles or clusters produced from the solid state are formed sufficiently small that they only contain a single analyte ion that is released upon complete matrix sublimation. Taylor cone formation in solids seems to be precluded. Without a mechanism for charge concentration, the low energy in charged matrix:analyte particles/clusters produced in MAIV may exclude ion evaporation so that only neutral matrix is lost in the desolvation process, thus leading to highly charged analyte ions. This is opposed to laser ablation where the thermal energy supplied to the particles likely promotes loss of charge through ion evaporation and thus favors low charge state ions [98]. A mechanism involving charged clusters has been proposed for MALDI [21–28]. We have previously suggested that charged droplets or particles are common to all of the new ionization methods, as well as to ESI and MALDI [20]. However, producing charged particles without energy input as in MAIV (Scheme 2, vertical transition) is surprising, and the mechanism whereby solid particles, in some cases highly charged, are produced containing only a single analyte ion is still unknown. While the necessary transitions from the solid and liquid state to gas-phase ions are different, as indicated in the phase diagram in Scheme 2 (horizontal transition of ‘evaporation’: ESI, MALDI, and *inlet* ionization; vertical transitions of sublimation: MAIV and likely LSIV), the final stages in producing the bare ions are related or the same. It is possible that the larger particles/droplets form the highly charged ions (ESI, LSIV, MAIV) and smaller particles/clusters (MALDI) form the singly charged ions. The more energetic the process, the more likely singly charged ions are observed (Scheme 3).

In addition to the proposal above, there are other potential mechanisms for the MAI process. One option is to fit the ionization mechanism into a currently accepted model. A possibility is that solvent included in the matrix with dissolved analyte might spray from cracks in the matrix when heated or exposed to vacuum. Here, charge might still be generated by the triboluminescent process and transferred to the expanding solvent droplets. Attempts to fully dry the matrix:analyte have been difficult because the matrix sublimes and included solvent is difficult to remove in the absence of heat and vacuum. Residual water in matrix:analyte samples has been discussed in MALDI and an excellent summary is provided in reference [78]. An alternate possibility is that the process somewhat resembles FD [42]. This may involve creation of a surface charge that provides a repulsive force to help remove the analyte ion from the surface. Because highly charged ions of proteins have not previously been observed by FD, and even desorption of relatively small ions required considerable heat, if the MAI process is related to FD, it would seem that sublimation of the matrix surrounding the analyte ion may be an additional requirement to release the analyte ion into the gas phase. Based on a considerable body of work, bare multiply charged gas-phase ions are not likely to be formed by such a process; however, it could be a mechanism for release of charged matrix:analyte clusters. Finally, the importance of the proper matrix, temperature, and pressure may suggest an unusual behavior associated with the matrix phase diagram (Scheme 2), for example, ion formation at the triple point. A number of experiments related to the importance of solid or liquid states of the matrix, as well as pH, have not yet provided clear mechanistic insights [19, 20, 82, 106, 119, 120]. It is likely that the mechanism has aspects in common with other matrix-assisted ionization (MAI) processes (Scheme 3).

### Relationship of the Novel Ionization Processes to Established Ionization Methods

Paving the way for the discovery of MAIV were a number of discoveries that did not fit conventional models. A common criticism received was that this is nothing new. We have been told that LSII is atmospheric pressure MALDI, LSIV is vacuum MALDI, and SAI is thermospray or sonic spray. Even MAII and MAIV (or simply MAI, Supplementary Table S1 and Scheme 3) have been called MA**LD**I even though no LD is required. Initially, these comments were a source of frustration, but over time we have come to believe they have substance, although likely viewed from a different perspective. Had MAI been discovered first, MALDI would then be a subset of MAI. Most likely, in MALDI, the laser plays some role in the ionization process, after all, LDI achieves ionization of compounds up to approximately 2 kDa without use of a matrix, and up to 7 kDa for volatile compounds [46–49, 121, 122]. Of course, with LDI as with, for example, plasma desorption, it is not clear that ionization of nonvolatile compounds did not involve a matrix, possibly included solvent [4]. However, in addition to our demonstration of ionization using a vacuum MALDI source without need of a laser [15, 16], Sugiyama and coworkers recently demonstrated that similar mass spectra to those observed using MALDI were obtained for proteins on a vacuum MALDI-TOF mass spectrometer for BSA using a temperature spike rather than a laser pulse [123]. Clearly, the ability to produce MALDI-like mass spectra from a protein with application of only thermal energy is further proof that photoionization is not a requirement in MALDI [20]. Thus, vacuum MALDI as well as LSIV can be viewed as MAI initiated with a laser (Scheme 3, right side). Of course, such a statement is controversial and needs to be tempered by the likelihood that there are ions observed in the MALDI process that are generated by photoionization processes (e.g., radical cations). Differences in charge states observed between the various ionization methods seem to point to a relationship of charge state to energy input (heat) as well as pressure (vacuum), rather than completely different ion formation mechanisms. Those that are capable of forming the ESI-like charge states and ion abundances are proposed to proceed via evaporation/sublimation of droplets/clusters [20].

The nearly identical mass spectra observed with MAI, LSI, SAI, and ESI suggests a mechanistic link between these ionization processes (Scheme 3, left side). Combining this with no obvious chemical structural features necessary for matrix assistance [19, 106], it seems probable that one necessary function of a matrix is its ability to solvate analyte. Otherwise, we should observe more multimers in the ionization process. Interestingly, ESI shows a significantly higher degree of aggregation than MAI [119]. Solvents, then, have this important characteristic of a matrix [110].

Numerous experiments suggest that the first step in forming gas-phase ions, especially from nonvolatile compounds, is to produce gas-phase charged droplets/particles/clusters by some charge separation process that can be envisioned to be one or two subsequent steps. Using a voltage, as in ESI, to achieve charge separation is an efficient process, but in nature, charge separation processes are common (e.g., thunderstorms) [11, 110]. The subsequent step is to remove the matrix/solvent to release the gas-phase analyte ions. The mechanistic implications of MAI may therefore encompass not only MAII, MAIV, LSII, and LSIV (Supplementary Table S1), but possibly SAI, ESI, FAB, MALDI, and thermospray (Scheme 3), and is thus of importance, even if MAI was not analytically useful. However, because MAI is highly sensitive, producing full scan mass spectra of ubiquitin from 1 fmol of analyte [124], and operates over a wide range of subatmospheric pressures without requiring a laser, voltage, or applied heat to produce gas-phase ions from low- and high-mass as well as volatile and nonvolatile compounds, the potential applications for analyses by MS appear endless, as my students say.

## Is there Room for Something New?

Examples of potential applications for MAI are provided in the Supplemental document. Readers interested in a more in-depth coverage of application areas using the new ionization methods from solids and solutions are referred to references [55, 56, 83, 84, 93, 105, 107, 125–130]. Here, a provocative section is provided in keeping with the purpose of *Critical Insights* articles.

Many years of effort have gone into improving ESI, MALDI, and APCI with excellent success [21–28, 35–41, 59, 131], but as these methods mature, it can be expected that new discoveries will become increasingly incremental. There are no doubt lessons to be learned from the new and currently, to a large extent, “magic” ionization presented here that will impact ionization in MS and possibly beyond. *Critical insights* are needed to understand not only the mechanism of the new ionization processes but also what this knowledge will teach us concerning established ionization processes and how to make improvements with a new and broadened vision. Obtaining a better understanding of MAI may also uncover entirely new applications for MS.

It should be evident that the new ionization processes are not fully explained by traditional MS wisdom. I personally love ‘the Loo’s’ *Critical Insights* in *JASMS*, as it is an exceptional example providing evidence of our limited and incomplete understanding of even the ESI mechanism [132]. While my group made some unexpected discoveries in the field of MS, and worked together with key collaborators to understand and apply this new technology, there is plenty left to understand and, no doubt, discover. Rather than get hung up on the acronyms used to describe the novel ionization methods (Supplementary Table S1), or whether they are thermospray or MALDI, it would be a logical step forward to focus on the process and subsequent results and how it may help the community to move the field forward.

While it is conceivable that many other new MAIV matrices might be discovered [102], the current challenges with this ionization technology in conjunction with a laser are to desolvate matrix clusters at the ‘right time’; that is not ‘too early’ because the matrix is sufficiently volatile to cause ionization prior to using a laser (MAIV) [133], or ‘too late’ because the matrix is not sufficiently volatile to cause ionization after the source region and during flight through the mass analyzer [17–20, 60, 61, 82], both limiting the full potential of LSI in terms of sensitivity and applicable mass range. A great deal of effort has gone into desolvation with ESI because it increases the sensitivity and aids in producing a steady ion current. No effort has gone into desolvation in vacuum MALDI until recently [20, 134, 135], but so far as charged clusters are involved, better desolvation should enhance ion abundance, especially of multiply charged ions. Of course, this is a challenge under high vacuum conditions, but lasers, microwaves, and collisions might enhance desolvation. We succeeded by using matrices that sublime or evaporate under vacuum to help desolvation, but there are undoubtedly additional means for achieving similar results for less volatile matrices without application of heat. The desolvation process is dependent on pressure as well as temperature, and high vacuum is problematic, possibly because there are too few collisions with gaseous molecules to counteract evaporative cooling. Ion source modifications that enhance not only charge separation but desolvation promises to further increase the sensitivity of the new ionization methods. It is interesting to note that MALDI matrices have been optimized for sufficient *stability* in vacuum [79, 80] while LSI and MAI matrices have been discovered by searching for increased *volatility* [15–20, 64, 106].

To date, all of the novel ionization processes have been accomplished on instruments designed for ESI or MALDI. Yet equivalent, and often better sensitivity [9, 10, 15, 16, 82, 104–108, 124, 136–139] and mass resolution [57, 58, 64, 67, 69, 84, 92, 106, 130] has been obtained by the new methods. Designing an instrument and inlet system specifically for the purpose of the new ionization methods will potentially provide even greater sensitivity. The new technology can be anticipated to be applicable to: (1) rapid surface analyses and imaging of small *and* large nonvolatile compounds at high spatial resolution directly from atmospheric pressure using *any* low and high performance mass spectrometer with and without the use of a laser [15, 16, 54–58, 61–65, 69, 83, 84, 91–94, 125, 126, 130, 133]; (2) advanced fragmentation technology based on collision induced dissociation, electron transfer dissociation, and/or potentially electron capture dissociation of abundant highly charged ions directly from surfaces [15, 16, 19, 20, 56, 58, 69, 93, 105, 125, 126, 130]; (3) advanced ion mobility measurements [17, 20, 62, 63, 83, 105, 108, 119, 125, 126] not only to enhance the analytical capabilities of the solvent-free gas-phase separation but to perform cross-section analyses directly from the native surface and not from worked up solutions that may or may not have anything to do with, e.g., the membrane protein conformation in the lipid bilayer, which is important for drug targeting; (4) portability and simplicity of use because only the vacuum of a mass spectrometer is needed in conjunction with embedded obstacle(s) to drive charge separation and desolvation [19, 20, 60–62, 82, 119]. While only the future will show if any of these challenges will be met, at least at this current stage, these are real opportunities to advance the field of MS.

## Conclusion and Hope

Hopefully, we have made a dent in understanding fundamentals and defining some potential application areas of the new ionization technologies. NSF has funded a university startup company (MS, LLC) through an STTR Phase I grant, which should allow more groups to use the technologies and provide new applications, clever new experiments, and fundamental knowledge, similar to the groups of, for example, Beauchamp, Caprioli, Clemmer, Cody, Cooks, Cramer, Isailovic, Kostiainen, Li, McEwen, Moskovets, Motoyama, Murray, Pergantis, Russell, and Zenobi [9, 10, 27, 28, 84, 91, 93, 95–97, 111, 112, 114, 123, 124, 134–149], to help drive this technology to the next level.

We experienced some headwind from some reviewers but, as Ellen Inutan said after she was awarded her Ph.D., “We wouldn’t have worked this hard and be where we are now if we didn’t receive criticism.” It has been an incredible journey and it has been a joy to watch students in my lab have a great learning experience and fun at the same time! I will end with the quote attributed to Mantak Chia: “The single most important point to remember about polarity is that *yin* and *yang* energies are not separate energies; they are one and the same energy, but with two different charges.”

## Electronic supplementary material

ESM 1 (PDF 653 kb)

ESM 2 Movie Clip 1_LSII_LTQ Velos (MP4 26770 kb)

ESM 3 Movie Clip 2_MAII_LTQ Velos (MP4 9152 kb)

ESM 4 Movie Clip 3_SAII_LTQ Velos (MP4 56866 kb)

ESM 5 Movie Clip 4_SAII _automated (MOV 2149 kb)

ESM 6 Movie Clip 5_SAIV_LTQ Velos (WMV 2022 kb)

ESM 7 Movie Clip 6_MAIV_SYNAPT G2 (MP4 155184 kb)

ESM 8 Movie Clip 7_MAIV_QDa (MP4 51995 kb)

ESM 9 Movie Clip 8_MAIV_prototype platform (WMV 37495 kb)
