# Supplementary material for: "Magic" Ionization Mass Spectrometry
Source: J Am Soc Mass Spectrom. 2015 Oct 20;27:4–21. doi: 10.1007/s13361-015-1253-4 (PMC4686549; doi:10.1007/s13361-015-1253-4)
Supplement: Supplementary file 1 — (PDF 653 kb) [file 13361_2015_1253_MOESM1_ESM.pdf]

Supplemental Material

“Magic” Ionization Mass Spectrometry

Sarah Trimpin[1,2,3]

[1] *Department of Chemistry, Wayne State University, Detroit, MI 48202*

[2] *Cardiovascular Research Institute, Wayne State University School of Medicine, Detroit, MI 48201*

[3] *MSTM, LLC, Newark, DE 19711*

Corresponding author: strimpin@chem.wayne.edu

1. Introduction to New Ionization Methods and Their Acronyms

Brief descriptions of the ionization methods for use in mass spectrometry (MS) discussed in this *Critical Insights* article are provided in **Table S1** and visualized in **Figure S1.** Short movie clips uploaded to the Supplemental information provide further insight into the new ionization processes. The sequence of the discoveries begin with laserspray ionization *inlet* (**Figure S1a-c**; **Supplementary Movie Clip**) and progresses through matrix-assisted ionization *inlet* (**Figure S1d**; **Supplementary Movie Clip**), laserspray ionization *vacuum*, solvent-assisted ionization *inlet* (**Figure S1e**; **Supplementary Movie Clips**), and finally matrix-assisted ionization *vacuum* (**Figure S1f**; **Supplementary Movie Clips**). These methods in **Table S1** and **Figure S1** produce ions from either the solid or solution states through the application of high voltage (ESI) or by laser ablation (MALDI), or by application of temperature and/or pressure, as in the methods here termed laserspray ionization (LSI) [1-6], matrix-assisted ionization (MAI) [7-10], and solvent-assisted ionization (SAI) [11-15].

Table S1: General descriptions of LSI, MAI, SAI and their relationships categorized into *inlet* and *vacuum* ionization.

	***Inlet* Ionization**<br>Matrix/analyte introduced from AP to a heated inlet first producing charged matrix/analyte particles followed by <u>evaporation</u>/sublimation of the matrix to produce analyte ions.	***Vacuum* Ionization**<br>Matrix/analyte is placed directly into vacuum and produces analyte ions without a heated inlet. Charged matrix/analyte particles and evaporation/<u>sublimation</u> are believed to be involved to produce analyte ions.
**Laserspray Ionization (LSI)**<br>Analyte in a solid matrix is irradiated by a laser pulse.	**Laserspray Ionization *Inlet* (LSII)**<br>Laser ablation is at AP. Matrix:analyte enters the <u>heated inlet</u> of the API mass spectrometer. (Laser can be aligned in transmission or reflection geometry)	**Laserspray Ionization *Vacuum* (LSIV)**<br>Laser ablation of matrix:analyte occurs in vacuum and produces ESI-like charge states. (laser fluence prefers to be <u>*low*</u>)
**Matrix-Assisted Ionization (MAI)**<br>Analyte in a solid matrix is introduced to the vacuum of the mass spectrometer.	**Matrix-Assisted Ionization *Inlet* (MAII)**<br>Matrix:analyte is introduced into the <u>heated inlet</u> of the API mass spectrometer.	**Matrix-Assisted Ionization *Vacuum* (MAIV)**<br>Matrix:analyte is placed into subatmospheric pressure and produces analyte ions spontaneously without the application of heat.
**Solvent-Assisted Ionization (SAI)**<br>A solution containing analyte is introduced to the vacuum of the mass spectrometer.	**Solvent-Assisted Ionization *Inlet* (SAII)**<br>Solvent:analyte is introduced into the <u>heated inlet</u> of the API mass spectrometer.	**Solvent-Assisted Ionization *Vacuum* (SAIV)**<br>Solvent:analyte is placed in the vacuum of the mass spectrometer frozen and produces ions spontaneously.

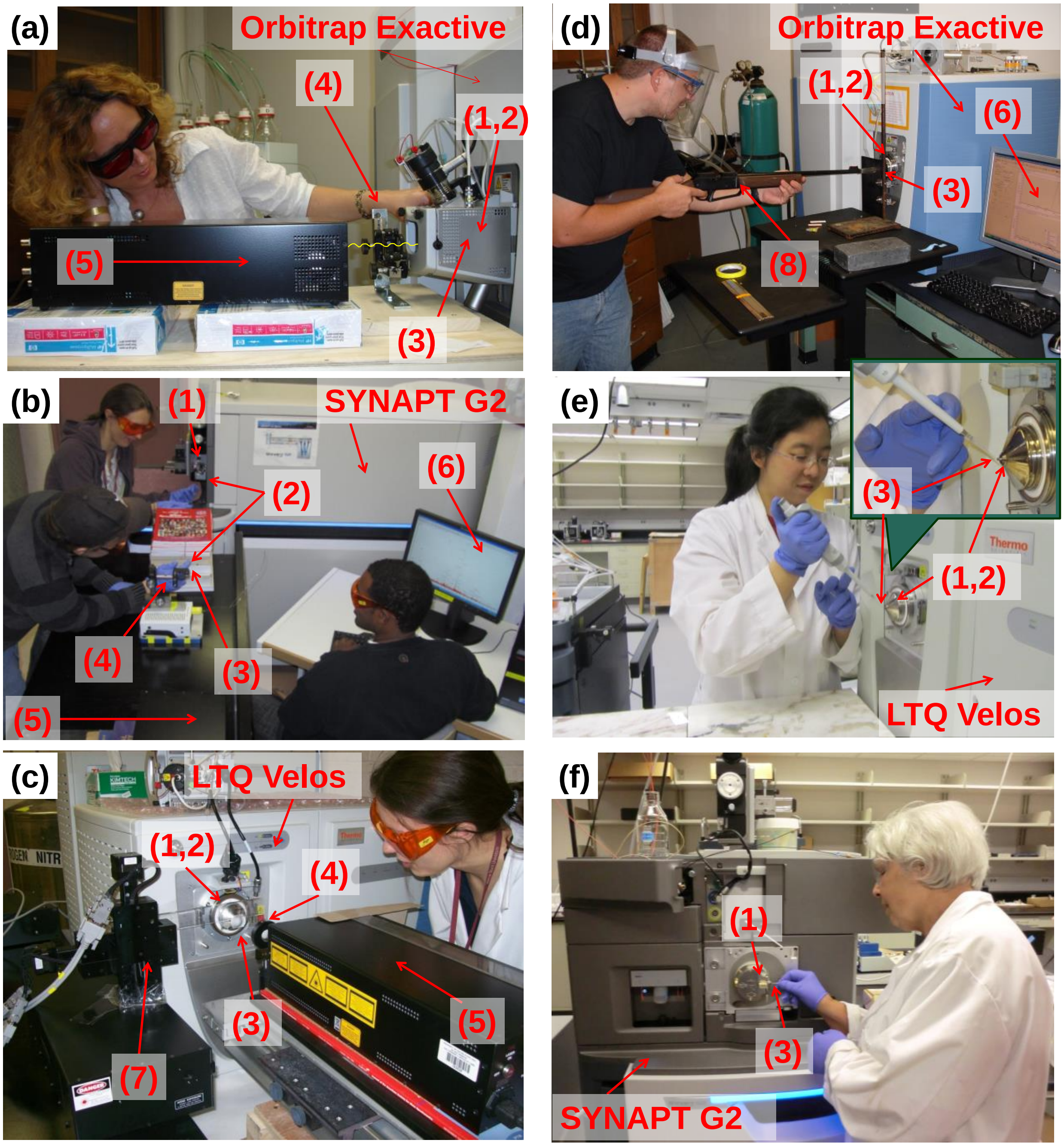

Figure S1: Evolution of new ionization processes: (**a**) first LSI experiments, (**b**) ions are created through the use of a 1-m long inlet tube without the need of additional heat suggested the importance of collisions with surfaces, (**c**) first transmission geometry LSI imaging successes, (**d**) exemplifies that a laser is not needed, (**e**) illustrates that the matrix can be a solvent, and (**f**) that anyone can perform MAI (see **Figure S2**). (**1**) inlet, (**2**) desolvation tube, (**3**) matrix:analyte sample, (**4**) focusing lens, (**5**) laser, (**6**) monitor with mass spectral results. Modified from Figure 2, with permission from Trimpin et al. [1]

A simple phase diagram from a MS perspective is shown in **Scheme 2** (main text) as a means to describe similarities and differences between more traditional ionization methods and those discussed here. The fundamentals driving the new ionization processes [16, 17], as they are currently understood, are discussed in this *Critical Insights* paper. Without a doubt, there is some 'hand-waving' involved. In understanding the fundamentals of the new ionization processes [18, 19], how the sample is introduced to the mass spectrometer can be ignored (**Figure S1**), focusing instead on how the formation of gas-phase ions depends on *temperature* and *pressure* (**Scheme 2**, main text) [1-9, 11-14, 16, 17]. Ignoring sample introduction methods is reasonable because the results are essentially the same, irrespective of how the matrix:analyte sample, solid or liquid, is presented to the mass spectrometer. That is, ions having charge states and abundances similar to ESI are observed, irrespective of whether the physical state is solid or solution. The similar charge states make the new methods potentially applicable to any mass spectrometer designed for ESI [1-23]. The new methods have cost advantages as the traditional ion source is not necessary, nor are high voltage supplies, lasers, or nebulizing/desolvation gases.

2. Serendipity was Our Best Friend in the Discovery of an Unknown Ionization Process

Early Lessons to Harbor Healthy Skepticism

Similar to most young students, in my early days learning about MS in class and later with research on Alzheimer's Disease during my 'Diplom' studies, I believed that ionization mechanisms were known truths. In ESI, there were two means of forming gas-phase ions, the charge residue [24] and ion evaporation models [25], and in MALDI, the laser had two functions, to evaporate the matrix leaving isolated neutral molecules suspended in the gas phase and to produce gas-phase reactant ions by photoionization [26, 27]. This sense of understanding did not last long. For me as a young scientist, 'the problem' started when interviewing for a PhD position at the Max-Planck-Institute (MPI) for Polymer Research in 1998 when I was told they wanted to analyze insoluble materials, potentially using MALDI-time-of-flight (TOF)-MS. From my just over one year of experience in MS, this idea went against the principles of MALDI that I believed at the time. However, the scientist at the MPI had much less reverence for the prevalent ionization models, especially regarding MALDI. At the time, I thought it a challenge and potentially an opportunity for something new. During my PhD, I learned that models don't necessarily represent reality.

My project was to develop a MALDI sample preparation approach for insoluble compounds that precluded the use of a solvent to isolate individual molecules in a matrix. I had been taught that, especially for nonvolatile compounds, incorporation within the matrix from solution was a requirement for successful ionization. A result of my research was the first *insoluble* macromolecules ionized by MALDI using solvent-free sample preparation [28]. It took an institutional effort to find the proper sample because most 'insoluble materials' are soluble in certain solution conditions [29]. After soxhlet extraction in hot toluene for 4 days, a solid residue remained and showed discernable higher mass components relative to the soluble fraction [28]. The ability to ionize the insoluble fraction using solvent-free MALDI seemed to be incompatible with the belief that analyte must be incorporated into the matrix during crystallization from solution for matrix-assistance to occur in MALDI [30]. Instead, these results seemed to suggest that incorporation of analyte into the matrix was not required in solvent-free MALDI, but that close contact is sufficient [30]. Hillenkamp, Karas, and coworkers proposed that true matrix assistance is required to produce ions from compounds exceeding 30,000 Da molecular weight, and this is only achieved by incorporation of analyte in the matrix [31]. Contrary, solvent-free MALDI was reported for synthetic polymers up to ~100 kDa as well as for bovine serum albumin, 66 kDa [32, 33]. The discovery that grinding the matrix with the analyte was applicable to insoluble compounds, and an efficient means of sample preparation for many compound types in MALDI [34, 35], left me dubious relative to our understanding of the MALDI ionization processes [30]. I am also now dubious of our proposal that intimate contact is sufficient [30], as it seems possible that with close contact incorporation of the analyte in the matrix may occur by melting, either during the grinding process or in molten droplets produced upon laser ablation [36, 37].

Regardless of the mechanistic aspects, it was quite gratifying to see the solvent-free sample preparation approach I had worked out during my Ph.D. developed further by Scott Hanton at Air Products, and his approach used extensively at, for example, DuPont [38, 39]. There are now 124 papers in which a simple solvent-free MALDI approach [32] is cited. Of course, as noted above, credits go to my Ph.D. advisors, Professor Müllen and Dr. Räder for suggesting this direction for my research. For me, the *Critical Insight* was that new developments can follow if one harbors a healthy case of skepticism.

3. The Discovery of the Unexpected

An important step for my personal development was 'bootcamp' as a research associate in Professor David Clemmer's lab. This was a time of intense learning about ion mobility spectrometry (IMS)-MS from David and his research group, but David also provided invaluable lessons on what is needed for success in the academic world. While in David's laboratory, I had the privilege of working with the late Professor J. Michael Walker (1950-2008), Department of Psychological and Brain Sciences, who was intrigued by the potential for MS to image endocannabinoids in rat brain to understand and combat human pain, especially that of chronic nature [40]. It was discussions with Michael that initiated my thinking about a means to accomplish transmission geometry tissue imaging directly from microscope slides at atmospheric pressure using MS. Transmission geometry laser alignment has been used with laser microprobe mass analyzer (LAMMA) and later with MALDI from vacuum conditions [41, 42]. An excellent summary on this topic, also called back-side illumination, was provided by Dreisewerd [43]. In 2007, imaging mass spectrometry in transmission geometry was an unaccomplished task. The plan was to increase the spatial resolution for imaging of endocannabinoids from brain sections using MS using a well-focused laser with backside illumination, while simplifying the approach through sample manipulations at atmospheric pressure. I was fully aware of the general opinion for the need to apply voltages to lift ions from surfaces both at vacuum and atmospheric pressure [44, 45]. However, I felt certain that laser ablation of an atmospheric pressure MALDI matrix from the backside would produce a jet of expanding matrix and ions which could be captured by the gas flowing into the inlet of a mass spectrometer. This atmospheric pressure transmission geometry imaging approach was a component in the research proposal I used during faculty interviews. These ideas, initiated by discussions with Michael, led to an unexpected discovery which is the heart of the *Critical Insights* paper.

4. Applications

We have pursued a comparison of different mass spectrometers with the 'old' and 'new' ionization methods relative to clinically relevant measurements. In quantification, MAI fared as well as ESI using an internal standard and surpasses MALDI [22]. MAI-MS accomplishes this in a fast, simple manner. To showcase this point, a retired nurse who happens to be my mother was invited to the lab to perform an experiment. She had never seen or touched a mass spectrometer. Her first ever attempt within minutes of training is depicted in **Figure S1f** using

bovine insulin and 3-NBN as matrix on the Waters SYNAPT G2 mass spectrometer without a source, heat, voltage, laser, or nebulizing gases applied to the inlet. In other words, the experiment was safe enough for my own mother. Multiply charged ions were observed, just like in ESI, but with minimal chemical background (**Figure S2**). This simple example demonstrates that untrained personnel can perform MAI with excellent results. Time requirements are mixing the matrix and analyte, as in MALDI, and only a few seconds to obtain the mass spectrum. Even the step of mixing the matrix and analyte has been circumvented in the MAI platform being produced by MS[TM] (Newark, DE) by having analyte and matrix mix in a syringe and then inserted into the atmospheric pressure inlet aperture (**Supplementary Movie**) [46].

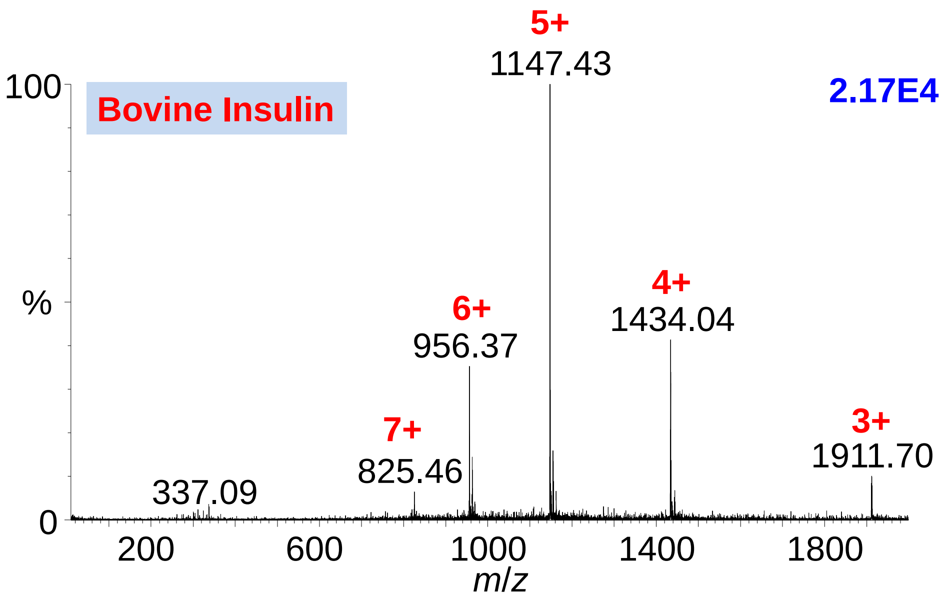

Figure S2: Retired nurse's first attempt of analyzing bovine insulin by MAI-MS using 3-NBN on the Waters SYNAPT G2 (**Figure S1f**). The red numbers indicate the charge state and the blue number in the top right corner indicates the ion abundance

In addition to operation on high end mass spectrometers, MAI is operational on a small portable mass spectrometer (Waters ACQUITY QDa Detector) for which the source housing interlocks were overridden and operated without sheath gas, high voltages, laser, and only moderate heat [23, 47]. The mass range (up to mass-to-charge 1250) of the mass spectrometer is extended, similar to ESI, because of the multiple charging. Rapid switching allows detection of positive and negatively charged analyte ions from the same analyte:matrix sample [23]. Of all mass spectrometers tested so far, only a single quadrupole, with the detector in-line with the quadrupole rods and the inlet, failed using 3-NBN as matrix, and this is hypothesized to be

because particles from the matrix reached the detector, causing oversaturation. The broad applicability to various mass spectrometers has been presented recently [23].

Drugs spiked in urine and drugs in the urine of a drug addicted newborn were readily detected using MAI [22, 23]. There is no sample cleanup and approximately 1 µL of analyte solution combined with the matrix is needed for exposure to the vacuum of the mass spectrometer. The measurements are accomplished within seconds. With ESI, these measurements are particularly difficult without a cleanup step because of the salty nature of the sample. Of course, sample preparation is similar to MALDI, but without the need of a laser and producing highly charged ions, MAI is compatible with high performance mass spectrometers (e.g., mass range, electron transfer dissociation or ETD, IMS, mass resolution). Because of the multiple charging, ETD of a fragile c-mycin modified peptide is accomplished (**Figure S3a**); neither the peptide nor labile functionality fragment [19]. Mass resolution values of ubiquitin were observed in the order 100,000 on a Quadrupole-Time-of-Flight (SYNAPT G2) using the MAI matrix 3-NBN ***22*** [48].

The MAI method also has utility for characterizing surface *monolayers* of iron (Fe^{III}), manganese (Mn^{III}) and other complexes, where MALDI and ESI produced either poor or no useful results [23, 49, 50]. The sensitivity and softness of MAI as a surface method is exceptional, suggesting that small matrix spots deposited on a surface can be used to obtain spatially resolved ions, albeit at low spatial resolution. Other synthetic materials are directly analyzed without any work-up procedure including carbohydrates, glycoconjugates, and polymer conjugates where again MALDI and ESI had difficulties or failed [23, 51-53]. Useful matrices for synthetic materials without basic functionality, were in these cases the solvent itself (SAI), 2-bromo-2-nitropropane-1,3-diol ***28***, and 2-methyl-2-nitropropane-1,3-diol ***29*** in the positive mode and 1,2-dicyanobenzene (1,2-DCB) ***25*** (**Scheme 1**, main text) in the positive and negative modes (especially for lipids) [11, 14, 23, 51-48]. The methyl-5-nitro-2-furoate 27 compound produces exceptionally high charge states and can be introduced as a solution without the need for a heated inlet tube [48] suggesting utility with liquid chromatography and high throughput applications similar to SAI [12, 23, 54]. For compounds that contain basic functionality such as drugs, peptides, and proteins, 3-NBN remains the preferred matrix for positive mode measurements [48].

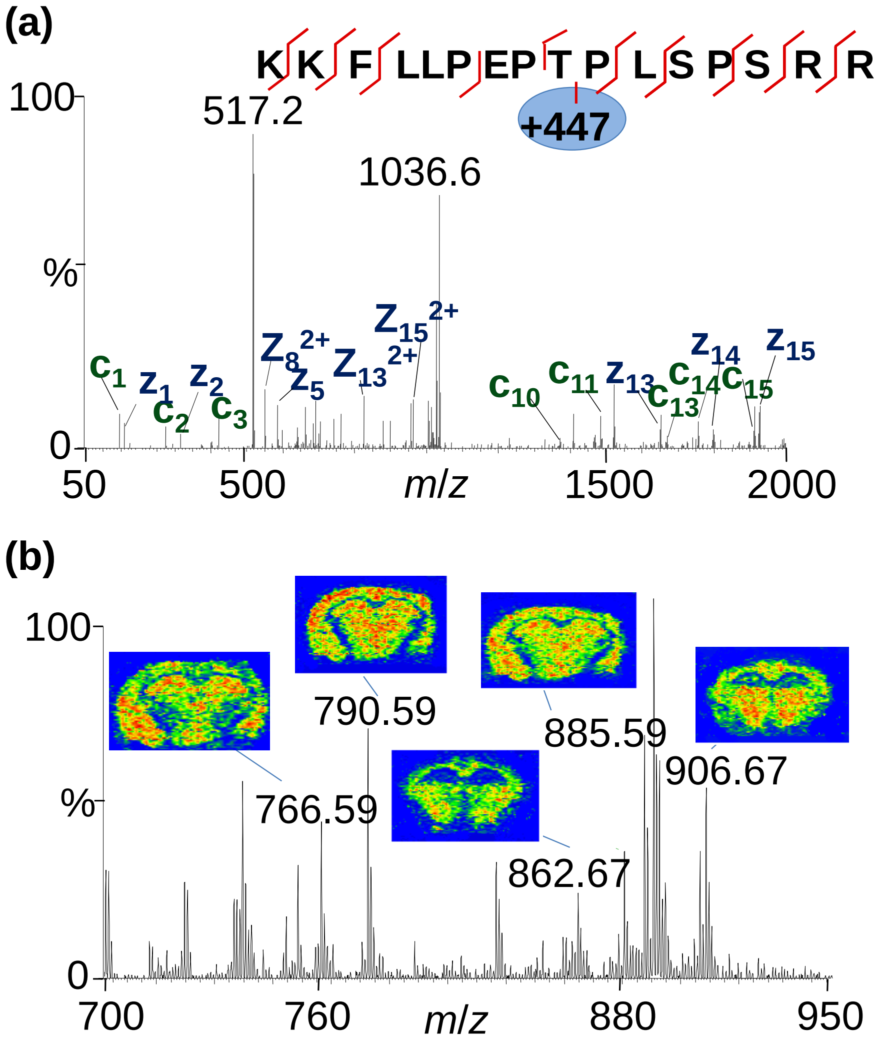

Figure S3: LSI: (**a**) C-Mycin labile peptide modification (~2.3 kDa) using 2,5-dihydroxybenzoic acid (2,5-DHB) ***5*** matrix on a LTQ and sequenced using ETD as published in ref. [19]; (**b**) mass spectrum and images of $[M–H]^-$ ions of lipids from mouse brain tissue with the laser aligned in transmission geometry. The 10 µm thick mouse brain tissue section was covered with the matrix 2,5-DHAP ***6*** solvent-free and acquired with an ion transfer capillary temperature of 450 °C on the LTQ Velos (see **Figure S1c**). Images of ions detected in the negative ionization mode are shown for $[M–H]^-$ with *m/z* of 766.59, 790.59, 862.67, 885.59 and 906.67. Modified from Figures S1 and Figure 4, with permission from Trimpin et al. [19] and Richards et al. [55].

Another application of MAI (**Scheme 3**, main text) is imaging. First experiments using atmospheric pressure backside, or transmission geometry, were performed (**Figure S1c**) in what we believed was a field-free MALDI experiment [1-3, 18, 19]. An example of this work is shown in **Figure S3b** [55]. A single laser shot produces a mass spectrum of, as examples, drugs, lipids, peptides, proteins, and synthetic polymers, increases the speed of systematically sampling a tissue surface in imaging studies [55-58] and improves the spatial resolution of the

analyses [1, 55, 59] using straightforward laser optics and geometry (**Figure S1a-c, Supplementary Movie**). Pre-deposited matrix applications to the glass surface prior to the tissue deposition typically enhances the ion abundance of the measurement and the quality of the imaging experiment [1, 55, 56, 59, 60]. More recently, other groups accomplished transmission geometry imaging using a laser [61 , 62]. Harron, et al. were the first to demonstrate transmission geometry LSI imaging of multiply charged protein ions using the MAI matrix, 3-nitrobenzonitrile (3-NBN) ***22***, mixed with 2,5-dihydroxyacetophenone (2,5-DHAP) ***6***, at ultra-high mass resolution using an Orbitrap Exactive and an inexpensive nitrogen laser, similar to **Figure S1a** but with the source housing removed [61]. Imaging of multiply charged ions were accomplished on the intermediate pressure source of the SYNAPT G2 with the laser aligned in reflection geometry using 2-nitrophloroglucinol (2-NPG) ***18*** matrix [5]. The use of ion mobility and ETD were demonstrated directly from tissue [59, 60]. Lingjun Li's group accomplished LSI tissue imaging using the vacuum source of an Orbitrap mass spectrometer obtaining MS/MS data for identification [63]. Caprioli's group imaged multiply charged ions from tissue using a Fourier Transform (FT) MS [64]. Working from vacuum, these groups and others [65] used 2-NPG as matrix [5].

5. Tribute to Learning and Teaching

It takes a community to raise a scientist and to be a part of new discoveries! I definitely have not worked in a 'vacuum' and I am grateful to all my teachers who have fueled my interest in science and 'prepared my mind' [66]. These include, chronologically, Professors Michael Przybylski (University of Konstanz), Klaus Müllen (MPI for Polymer Research), late Max Deinzer (OSU), Peter Spencer (OHSU), and David Clemmer (IU). I have been blessed to have met established scientists who have inspired and guided me in my decision making throughout my career. Professors late John B. Fenn (VCU), Fred W. McLafferty (Cornell U.), late J. Michael Walker (IU), Charles N. McEwen (USciences; thanks for proofreading this manuscript!), Brian Chait (Rockefeller U.), Ken Mackie (IU), Barbara J. Garrison (Penn State), Catherine Fenselau (UM), Michael L. Gross (UWash), David H. Russell (Texas A&M), Scott A. McLuckey (Purdue U.), and Donald F. Hunt (UVa) as well as Drs. Hans-Joachim Räder (MPI for Polymer Research), Barbara S. Larsen (DuPont), Alexander Makarov (Thermo), and Tim Riley (Waters), are examples.

On a personal note, about the time that I obtained my PhD degree equivalent from MPI, Professor John B. Fenn received a portion of the Nobel Prize in Chemistry for his work on ESI.

He and Fred McLafferty stood out in my mind as two of the giants in MS. As a postdoc, I was thrilled to be assigned to be John Fenn's driver during his visit to Oregon State University. To my amazement, he always remembered me when we were at the same conferences and even shared many academic life stories with me. These informative informal talks *very much taught me new aspects of healthy skepticism*. I first met Fred McLafferty at ASMS and over the years he has shown great kindness to me, and many other budding mass spectrometrists. Now my students are just as excited as I was when Fred came by their posters. I mention this because there have been a number of scientists in the MS community with exceptional credentials who have likewise mentored my development and *encouraged me*, both essential for success. Therefore the developments leading to this *Critical Insights* article is also their success.

I also have been blessed to have graduate and undergraduate students as well as postdocs who have been exceptional, and have become outstanding scientists and teachers. I would like to express my deepest gratitude to them for being *my* teacher (job well done for being kind and having an appetite for discovery and good science!). In chronological order: Dr. Ellen D. Inutan (U. Mindanao), S. Alexandru Cernat (Imperial Oil), Alicia L. Richards (U. Wisconsin), Christopher B. Lietz (U. Wisconsin), Dr. Beixi Wang (U. Michigan), Samantha M. Leach (DC Department of Forensic Sciences), Darrell D. Marshall (U. Nebraska), Corey D. Manly (U. Colorado Medical School), Corinne A. Lutomski (IU), Tarick J. El-Baba (IU), Daniel W. Woodall, Bryan M. Harless, Casey D. Foley, Jessica L. DeLeeuw, Zachary J. Devereaux, Shameemah M. Thawoos, Joshua L. Fischer, and Dr. Christian Reynolds. These co-workers especially have helped me learn valuable lessons about young scientists' potential, given the right environment and opportunities. Watching young scientists take ownership of their research, realize the excitement of each discovery, however small, become leaders in the laboratory, and finally, drive the direction of the lab is wonderful to observe.

I am still learning, and it is simply amazing to see the transformation of a(n) (under)graduate student into a young scientist as they become aware that their research has meaning and importance and that others are paying attention to their discoveries, such as this invitation (thank you Professor David H. Russell!) to contribute a *Critical Insights* Article to one of my students' most favored journals. Student participation in research is key for every lab, and one element of learning that is especially effective is participation at conferences and symposia, both at the graduate and undergraduate level. ASMS is superb for exactly this reason and I am extremely thankful to this organization! I would like to thank the ASMS attendees for being my, and my students', teachers by discussing with us aspects of this new ionization process over the past 5 years. There is much to be learned!

6. References

1. Trimpin, S., Herath, T.N., Inutan, E.D., Cernat, S.A., Wager-Miller, J., Mackie, K., Walker, J.M.: Field-free Transmission Geometry Atmospheric Pressure Matrix-assisted Laser Desorption/ionization for Rapid Analysis of Unadulterated Tissue Samples. Rapid Commun. Mass Spectrom. **23**, 3023–3027 (2009)
2. Trimpin, S., Inutan, E.D., Herath, T.N., McEwen, C.N.: Matrix-Assisted Laser Desorption/Ionization Mass Spectrometry Method for Selectively Producing Either Singly or Multiply Charged Molecular Ions. Anal. Chem. **82**, 11–15 (2010)
3. Trimpin, S., Inutan, E.D., Herath, T.N., McEwen, C.N.: Laserspray Ionization – A New Atmospheric Pressure MALDI Method for Producing Highly Charged Gas-Phase Ions of Peptides and Proteins Directly from Solid Solutions. Mol. Cell Proteomics **9**, 362–367 (2010)
4. Inutan, E.D., Wang, B., Trimpin, S.: Commercial Intermediate Pressure MALDI Ion Mobility Spectrometry Mass Spectrometer Capable of Producing Highly Charged Laserspray Ionization Ions. Anal. Chem. **83**, 678–684 (2010)
5. Trimpin, S., Ren, Y., Wang, B., Lietz, C.B., Richards, A.L., Marshall, D.D., Inutan, E.D.: Extending the Laserspray Ionization Concept to Produce Highly Charged Ions at High Vacuum on a Time-of-Flight Mass Analyzer. Anal. Chem. **83**, 5469–5475 (2011)
6. Lutomski, C.A., El-Baba, T.J., Inutan E.D., Manly, C.D., Trimpin, S.: Transmission Geometry Laserspray Ionization Vacuum Using an Atmospheric Pressure Inlet. Anal. Chem. **86**, 6208–6213 (2014).
7. McEwen, C.N., Pagnotti, V., Inutan, E.D., Trimpin, S.: A New Paradigm in Ionization: Multiply Charged Ion Formation from as Solid Matrix without a Laser or Voltage. Anal. Chem. **82**, 9164–9168 (2010)
8. Trimpin, S. Inutan, E.D.: Matrix Assisted Ionization in Vacuum, a Sensitive and Widely Applicable Ionization Method for Mass Spectrometry. J. Am. Soc. Mass Spectrom. **24**, 722–732 (2013)
9. Inutan, E.D., Trimpin, S.: Matrix Assisted Ionization Vacuum, a New Ionization Method for Biological Materials Analysis using Mass Spectrometry. Mol. Cell Proteomics **12**, 792–796 (2013)
10. Inutan, E.D., Wager-Miller, J., Narayan, S.B., Mackie, K., Trimpin, S.: The Potential for Clinical Applications using a New Ionization Method Combined with Ion Mobility Spectrometry-Mass Spectrometry. Int. J. Ion Mobility Spectrom. **16**, 145–159 (2013)
11. Pagnotti, V.S., Chubatyi, N.D., McEwen, C.N.: Solvent Assisted Inlet Ionization: An Ultrasensitive New Liquid Introduction Ionization Method for Mass Spectrometry. Anal. Chem. **83**, 3981–3985 (2011)
12. Wang, B., Inutan, E.D., Trimpin, S.: A New Approach to High Sensitivity Liquid Chromatography-Mass Spectrometry of Peptides using Nanoflow Solvent Assisted Inlet Ionization. J. Am. Soc. Mass Spectrom. **23**, 442–445 (2012)
13. Wang, B., Trimpin, S.: High Throughput Solvent Assisted Ionization Inlet (SAII) for Use in Mass Spectrometry. Anal. Chem. **86**, 1000–1006 (2014)
14. Pagnotti, V.S., Chakrabarty, S., Wang, B., Trimpin, S., McEwen, C.N.: Gas-Phase Ions Produced by Freezing Water or Methanol for Analysis Using Mass Spectrometry. Anal. Chem. **86**, 7343–7350 (2014)
15. Wang, B., Dearring, C.L., Wager-Miller, J., Mackie, K., Trimpin, S.: Drug Detection and Quantification Directly from Tissue using Novel Ionization Methods for Mass Spectrometry. Eur. J. Mass Spectrom., **21**, 201-210 (2015)

16. Lietz, C., Richards, A., Ren, Y., Trimpin, S.: Highs and Lows: Small to Large Protein Analysis with Matrix-Assisted Inlet Ionization (MAII) Techniques in Positive and Negative Ion Mode. 59th ASMS Conference, Denver, CO, June 4-9 (2011)
17. Trimpin, S.: Imaging Mass Spectrometry (MS) at Atmospheric and Intermediate Pressure using Laserspray Ionization (LSI). 59th ASMS Conference, Denver, CO, June 4-9 (2011)
18. Li, J., Inutan, E.D., Wang, B., Lietz, C.B., Green, D.R., Manly, C.D., Richards, A.L., Marshall, D.D., Lingenfelter, S., Ren, Y., Trimpin, S.: Matrix Assisted Ionization: New Aromatic and Non Aromatic Matrix Compounds Producing Multiply Charged Lipid, Peptide, and Protein Ions in the Positive and Negative Mode Observed Directly from Surfaces. J. Am. Soc. Mass Spectrom. **23**, 1625–1643 (2012)
19. Trimpin, S., Wang, B., Inutan, E.D., Li, J., Lietz, C.B., Pagnotti, V.S., Harron, A.F., Sardelis, D., McEwen, C.N.: A Mechanism for Ionization of Nonvolatile Compounds in Mass Spectrometry: Considerations from MALDI and Inlet Ionization. J. Am. Soc. Mass Spectrom. **23**, 1644–1660 (2012)
20. Trimpin, S., Inutan, E.D.: New Ionization Method for Rapid Analysis on Atmospheric Pressure Ionization Mass Spectrometers Requiring Only Vacuum and Matrix Assistance. Anal. Chem. **85**, 2005–2009 (2013)
21. Woodall, D.W., Wang, B., Inutan, E.D., Narayan, S.B., Trimpin, S.: High-throughput Characterization of Small and Large Molecules Using Only a Matrix and the Vacuum of a Mass Spectrometer. Anal. Chem. **87**, 4667–4674 (2015)
22. Chakrabarty, S., DeLeeuw, J.L., Woodall, D.W., Jooss, K., Narayan, S.B., Trimpin, S.: Reproducibility and Quantitation of Illicit Drugs using Matrix-Assisted Ionization (MAI) Mass Spectrometry. Anal. Chem. **87**, 8301-8306 (2015)
23. Trimpin, S., Reynolds, C.A., Thawoos, S., DeLeeuw, J.L., Devereaux, Z.J., Chakrabarty, S., Foley, C.D., Woodall, D.W., Fischer, J.L., Wang, B., Harless, B.M., Verani, C.N., Allen, M.J., Sanderson, T.H., Przyklenk, K., Narayan, S.B., Caruso, J.A., Stemmer, P.M.: Matrix-Assisted Ionization: Enhancing Mass Spectrometry through Proper Sampling Conditions on Small Portable to High Performance Mass Spectrometers. 63rd ASMS Conference on Mass Spectrometry and Allied Topics, St. Louis, MO, May 31-June 1 (2015)
24. Dole, M., Mack, L.L., Hines, R.L., Mobley, R.C., Ferguson, L.D., Alice, M.B.: Molecular Beams of Macroions. J. Chem. Phys. **49**, 2240–2249 (1968)
25. Iribarne, J.V., Thomson, B.A.: On the Evaporation of Small Ions from Charged Droplets. J. Chem. Phys. **64**, 2287–2294 (1976)
26. Karas, M., Hillenkamp, F.: Laser Desorption Ionization of Proteins with Molecular Masses Exceeding 10,000 Daltons. Anal. Chem. **60**, 2299–2301 (1988)
27. Ehring, H., Karas, M., Hillenkamp, F.: Role of Photoionization and Photochemistry in Ionization Processes of Organic Molecules and Relevance for Matrix-assisted Laser Desorption Ionization Mass Spectrometry. Org. Mass Spectrom. **27**, 472–480 (1992)
28. Trimpin, S., Grimsdale, A.C., Rader, H.J., Mullen, K.: Characterization of an Insoluble Poly(9,9-diphenyl-2,7-fluorene) by Solvent-free Sample Preparation for MALDI-TOF Mass Spectrometry. Anal. Chem. **74**, 3777–3782 (2002)
29. Przybilla, L., Brand, J.D., Yoshimura, K., Rader, H.J., Mullen, K.: MALDI-TOF mass spectrometry of insoluble giant polycyclic aromatic hydrocarbons by a new method of sample preparation. Anal. Chem. 72, 4591–4597 (2000)
30. Trimpin, S., Rader, H.J., Mullen, K.: Investigations of Theoretical Principles for MALDI-MS Derived from Solvent-free Sample Preparation - Part I. Preorganization. Int. J. Mass Spectrom. **253**, 13–21 (2006)
31. Gluckmann, M., Pfenninger, A., Kruger, R., Thierolf, M., Karas, M., Horneffer, V., Hillenkamp, F., Strupat, K.: Mechanisms in MALDI Analysis: Surface Interaction or Incorporation of Analytes? Int. J. Mass Spectrom. **210**, 121–132 (2001)

32. Trimpin, S., Rouhanipour, A., Az, R., Rader, H.J., Mullen, K.: New Aspects in Matrix-Assisted Laser Desorption/Ionization Time-of-Flight Mass Spectrometry: a Universal Solvent-Free Sample Preparation. Rapid Commun. Mass Spectrom. **15**, 1364–1373 (2001)
33. Trimpin, S., Deinzer, M.L.: Solvent-free MALDI-MS for the Analysis of Biological Samples via a Mini-ball Mill Approach. J. Am. Soc. Mass Spectrom. **16**, 542–547 (2005)
34. Trimpin, S., Keune, S., Räder, H.J., Müllen, K.: Solvent-free MALDI-MS: Developmental Improvements in the Reliability and the Potential of MALDI Analysis of Synthetic Polymers and Giant Organic Molecules. J. Am. Soc. Mass Spectrom. **17**, 661–671 (2006)
35. Trimpin, S., Deinzer, M.L.: Solvent-free MALDI-MS for the Analysis of Beta-amyloid Peptides via the Mini-ball Mill Approach: Qualitative and Quantitative Advances. J. Am. Soc. Mass Spectrom. **18**, 1533–1543 (2007)
36. McEwen, C.N., Trimpin, S.: An alternative ionization paradigm for atmospheric pressure mass spectrometry: Flying elephants from Trojan horses. Int. J. Mass Spectrom. **300**, 167–172 (2011)
37. Wang, B., Lietz, C.B., Inutan, E.D., Leach, S.M., Trimpin, S.: Producing Highly Charged Ions without Solvent Using Laserspray Ionization: A Total Solvent-Free Analysis Approach at Atmospheric Pressure. Anal. Chem. **83**, 4076–4084 (2011)
38. Hanton, S.D., Parees, D.M.: Extending the Solvent-free MALDI Sample Preparation Method. J. Am. Soc. Mass Spectrom. **16**, 90–93 (2005)
39. Trimpin, S., McEwen, C.N.: Multisample Preparation Methods for the Solvent-free MALDI-MS Analysis of Synthetic Polymers. J. Am. Soc. Mass Spectrom. **18**, 377–381 (2007)
40. Tsou, K., Brown, S., Sanudo-Pena, M.C., Mackie, K., Walker, J.M.: Immunohistochemical distribution of cannabinoid CB1 receptors in the rat central nervous system. Neuroscience **83**, 393–411 (1998)
41. Wechsung, R., Hillenkamp, F., Kaufmann, R., Nitsche, R.; Unsold, E., Vogt, H.: LAMMA – New Laser-Microscope-Mass Analyzer. Microscopica Acta **2**, 281–296 (1978)
42. Vertes, A., Balazs, L., Gijbels, R.: Matrix-assisted laser desorption of peptides in transmission geometry. Rapid Commun Mass Spectrom **4**, 263–266 (1990)
43. Dreisewerd, K.: The Desorption Process in MALDI. Chem. Rev. **103**, 395–425 (2003)
44. Vestal, M.L., Juhasz, P., Martin, S.A.: Delayed extraction matrix-assisted laser desorption time-of-flight mass spectrometry. Rapid Commun. Mass Spectrom. **9**, 1044–1050 (1995)
45. Laiko, V.V., Baldwin, M.A., Burlingame, A.L.: Atmospheric Pressure Matrix-Assisted Laser Desorption/Ionization Mass Spectrometry. Anal. Chem. **72**, 652–657 (2000)
46. MSTMsolutions.com
47. Trimpin, S., Lutomski, C., El-Baba, T., Wang, B., Imperial, L., Woodall, D., Kumar, R., Harless, B., Foley, C., Liu, C.W., Inutan, E: Surprising New Ionization Methods for Mass Spectrometry, Mechanistic Insights and Potential Practical Utility. 62nd ASMS Conference on Mass Spectrometry and Allied Topics, Baltimore, Maryland, June 15-19 (2014)
48. Trimpin, S., Lutomski, C.A., El-Baba, T.J., Woodall, D.W., Foley, C.D., Manly, C.D., Wang, B., Liu, C.W., Harless, B.M., Kumar, R., Imperial, L.F., Inutan, E.D.: Magic Matrices for Ionization in Mass Spectrometry. Int. J. Mass Spectrom. **377**, 532–545 (2015)
49. El-Baba, T.J., Wickramasinghe, L.D., Verani, C.N., Trimpin, S.: Characterization of Monolayer Films of Asymmetric Metallosurfactants by Matrix Assisted Ionization Vacuum Mass Spectrometry. 62nd ASMS Conference on Mass Spectrometry and Allied Topics, Baltimore, Maryland, June 15-19 (2014)
50. Wickramasinghe, L.D., Perera, M.M., Li, L., Mao, G.Z., Zhou, Z.X., Verani, C.N.: Rectification in Nanoscale Devices Based on an Asymmetric Five-Coordinate Iron(III) Phenolate Complex. Angew. Chem. **52**, 13346–13350 (2013)
51. Wang, B., Liao, G., Guo, Z., Trimpin, S.: Characterization of Carbohydrate-Monophosphoryl Lipid Conjugate Cancer Vaccine Candidates using Matrix Assisted Ionization Vacuum Mass

Spectrometry. 62nd ASMS Conference on Mass Spectrometry and Allied Topics, Baltimore, Maryland, June 15-19 (2014)
52. Fischer, J.L., Lutomski, C.A., El-Baba, T.J., Siriwardana-Mahanama, B.N., Weidner, S.M., Falkenhagen, J., Allen, M.J., Trimpin, S.: Characterization of a Europium-Containing 2-Arm Polymer Conjugate by Matrix-Assisted Ionization-Ion Mobility Spectrometry-Mass Spectrometry. J. Am. Soc. Mass. Spectrom. (2015) DOI: 10.1007/s13361-015-1233-8
53. Foley, C.D., Larsen, B.S., Trimpin S.: Application of Matrix-Assisted Ionization–Ion Mobility–Mass Spectrometry to Polymeric Surfaces directly from Natural Environments. 63rd ASMS Conference on Mass Spectrometry and Allied Topics, St. Louis, MO, May 31-June 1 (2015)
54. Wang, B., Trimpin, S.: High Throughput Solvent Assisted Ionization Inlet (SAII) for Use in Mass Spectrometry. Anal. Chem. **86**, 1000–1006 (2014)
55. Richards, A.L., Lietz, C.B., Wager-Miller, J., Mackie, K., Trimpin, S.: Imaging Mass Spectrometry in Transmission Geometry. Rapid Commun. Mass Spectrom. **25**, 815–820 (2011)
56. Richards, A.L., Lietz, C.B., Wager-Miller, J., Mackie, K., Trimpin, S.: Localization and Imaging of Gangliosides in Mouse Brain Tissue Sections by Laserspray Ionization Inlet. J. Lipid Res. **53**, 1390–1398 (2012)
57. Trimpin, S., Wang, B., Lietz, C.B., Marshall, D.D., Richards, A.L., Inutan, E.D.: New Ionization Processes and Applications for Use in Mass Spectrometry. Crit. Rev. Biochem. Mol. **48**, 409–429 (2013)
58. El-Baba, T.J., Lutomski, C.A., Wang, B., Inutan, E.D., Trimpin, S.: Toward High Spatial Resolution Sampling and Characterization of Biological Tissue Surfaces using Mass Spectrometry. Anal. Bioanal. Chem. **406**, 4053–4061 (2014)
59. Inutan, E.D., Richards, A.L., Wager-Miller, J., Mackie, K., McEwen, C.N., Trimpin, S.: Laserspray Ionization - A New Method for Protein Analysis Directly from Tissue at Atmospheric Pressure with Ultrahigh Mass Resolution and Electron Transfer Dissociation. Mol. Cell Proteomics **10**, 1–8 (2011)
60. Inutan, E.D., Wager-Miller, J., Mackie, K., Trimpin, S.: Laserspray Ionization Imaging of Multiply Charged Ions using a Commercial Vacuum MALDI Ion Source. Anal. Chem. **84**, 9079–9084 (2012)
61. Harron, A.F., Khoa, H., McEwen, C.N.: High mass resolution tissue imaging at atmospheric pressure using laserspray ionization mass spectrometry. Int. J. Mass Spectrom. **352**, 65–69 (2013)
62. Zavalin, A., Todd, E.M., Rawhouser, P.D., Yang, J., Norris, J.L., Caprioli, R.M.: Direct imaging of single cells and tissue at subcellular spatial resolution using transmission geometry MALDI MS. J. Mass Spectrom. **47**,1473–1481 (2012)
63. Chen, B., Lietz, C.B., Li, L.: In Situ Characterization of Proteins Using Laserspray Ionization on a High-Performance MALDI-LTQ-Orbitrap Mass Spectrometer. J. Am. Soc. Mass Spectrom. **25**, 2177–2180 (2014)
64. Rizzo, D.G., Spraggins, J.M., Rose, K.L., Caprioli, R.M.: Imaging and Accurate Mass identification of Intact Proteins Abave 10 kDa using Multily Charged Ions and High Resolution MS. 61st ASMS Conference on Mass Spectrometry and Allied Topics, Minneapolis, Minnesota, June 9-13 (2013)
65. Fernandez-Iglesias, N., Bettmer, J.: Synthesis, purification and mass spectrometric characterisation of a fluorescent Au-9@BSA nanocluster and its enzymatic digestion by trypsin. Nanoscale **6**, 716–721 (2014)
66. Louis Pasteur: Dans les champs de l'observation le hasard ne favorise que les esprits préparés. Lecture, University of Lille, 7 December (1854)
